# Development and Validation of a Novel Model to Predict Regional Lymph Node Metastasis in Patients With Hepatocellular Carcinoma

**DOI:** 10.3389/fonc.2022.835957

**Published:** 2022-02-11

**Authors:** Xiaoyuan Chen, Yiwei Lu, Xiaoli Shi, Guoyong Han, Jie Zhao, Yun Gao, Xuehao Wang

**Affiliations:** ^1^ School of Medicine, Southeast University, Nanjing, China; ^2^ Hepatobiliary Center, The First Affiliated Hospital of Nanjing Medical University, Nanjing, China; ^3^ Key Laboratory of Liver Transplantation, Chinese Academy of Medical Sciences, Nanjing, China; ^4^ NHC Key Laboratory of Living Donor Liver Transplantation (Nanjing Medical University), Nanjing, China; ^5^ Department of General Surgery, The Affiliated Jiangning Hospital of Nanjing Medical University, Nanjing, China

**Keywords:** hepatocellular carcinoma, lymph node metastasis, lymph node dissection, nomogram, the SEER program

## Abstract

**Background:**

The evaluation of the nodal status of hepatocellular carcinoma (HCC) is a classic but controversial topic. This study aimed to investigate the incidence of lymph node metastasis (LNM), explore the role of lymph node dissection (LND), and develop and validate a novel model to predict LNM in patients with HCC, not other specified (NOS).

**Methods:**

The study cohort was taken from the Surveillance, Epidemiology, and End Results database. The annual percent change (APC) was calculated using the Joinpoint regression. Survival analyses adopted the competing risk model. The nomogram was constructed based on the least absolute shrinkage and selection operator (LASSO) logistic regression algorithm and validated by calibration curves. The area under the receiver operating characteristic curve (AUROC) was obtained to compare prognostic performance. Decision curve and clinical impact curve analyses were introduced to examine the clinical value of the models.

**Results:**

A total of 8,829 patients were finally enrolled in this study, and 1,346 (15.2%) patients received LND. The LND rate showed no noticeable fluctuation in the last decade, with an APC of 0.5% (P=0.593). LNM was identified in 56 (4.2%) patients and confirmed an independent prognostic factor of HCC patients (P=0.005). There were 2,497 lymph nodes retrieved, and 93 (3.7%) of them were positive. After propensity score matching, LND indicated no direct oncologic benefit and did not worsen competing risks. Moreover, an increased number of lymph nodes retrieved could not improve prognoses. 1,346 patients with LND were further randomly divided into the training and validation sets with the ratio of 1:1. Race, tumor size, clinical T stage, extrahepatic bile duct invasion, and tumor grade were independent risk factors for LNM. The constructed model was well calibrated and showed good discrimination power and net benefits in clinical practice.

**Conclusion:**

LNM is an independent prognostic factor in HCC, but routine LND seems to be unnecessary in HCC patients. The constructed model could predict the presence of LNM in HCC patients with good performance, which is meaningful to patient stratification and individual treatment strategies optimization.

## Introduction

Hepatocellular carcinoma (HCC) accounts for approximately 85%-90% of primary liver cancer and has been a heavy global health burden in the past few decades. According to a US national survey, HCC is the 5^th^ and 7^th^ cancer type of the estimated new cancer deaths for males and females, respectively (1–6). Due to cytological variants, HCC has several rare pathological subtypes, including fibrolamellar, clear cell, spindle cell, scirrhous and pleomorphic. Considering that these subtypes have unique clinicopathological features, the main focus of this study is hepatocellular carcinoma, not otherwise specified (NOS) (7, 8).

The lymph node is the second most common destination of extrahepatic metastasis in HCC (9). Compared with hematogenous metastasis, lymph node metastasis (LNM) is a rare but equally vital prognostic factor. The reported incidence of LNM varies, from 1.2% to 15.3% in clinical practice and up to 30.3% in autopsy (10–18). Because of the dismal prognoses, patients with LNM would be considered to have a systemic disease and classified into advanced stages, including stage IVa in the American Joint Committee on Cancer (AJCC) staging system, stage IIIb in the China Liver Cancer (CNLC) staging system and stage C in the Barcelona Clinic Liver Cancer (BCLC) staging system (6, 19). Nevertheless, the value of lymph node dissection (LND) in HCC still remains controversial. Several researchers have supported routine LND for outcome improvement, complication prevention, and comprehensive evaluation (20–22). Some other studies demonstrated no benefit of LND because of the similar survival, low incidence of LNM, and potentially increased postoperative morbidity (10, 11, 23–25). Another concern is how to accurately evaluate nodal status before surgery, which is important for decision-making of surgical strategies. Nowadays, the most common method to judge the presence of LNM is based on the size of lymph nodes on CT imaging. However, Ercolani et al. reported that the diagnostic accuracy of this method was only 46.1% (22).

It is difficult for a single institute to obtain enough positive research cases due to the low incidence of LNM in HCC (23). Under such circumstances, this study enrolled 8,829 patients who underwent surgery from a national high-volume database to investigate the prevalence of LNM, explore the role of LND, and develop and validate a novel model to stratify the risks of LNM in HCC patients.

## Patients and Methods

### Patients

**Figure 1:**
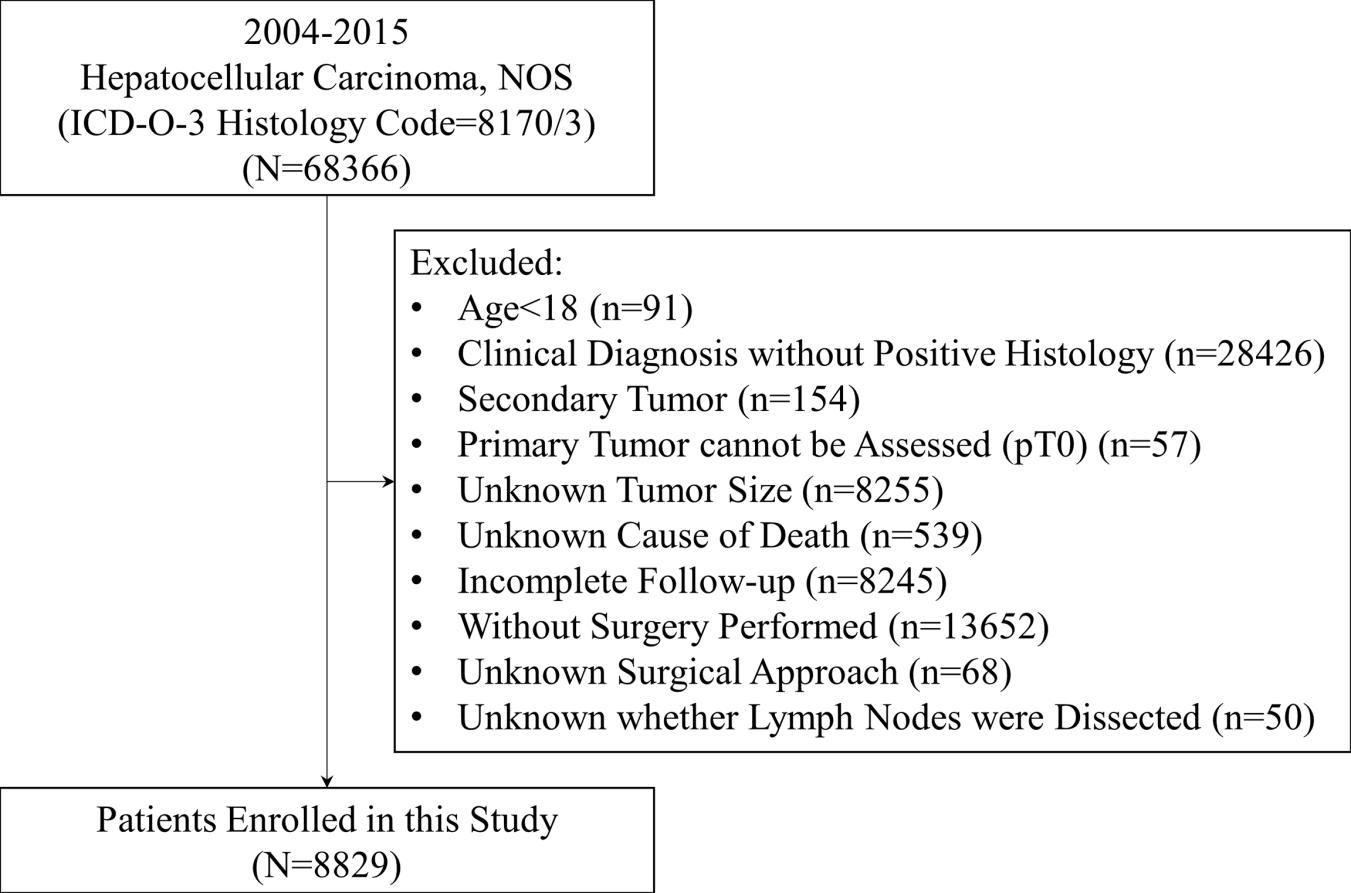
Stepwise extraction process from the Surveillance, Epidemiology, and End Results database. NOS, Not otherwise specified; ICD, International classification of diseases.

This study is a retrospective cohort study. Patients diagnosed with HCC, NOS (ICD-O-3 Histology Code=8170/3) from 2004 to 2015 were extracted from the Surveillance, Epidemiology, and End Results (SEER) Research Database (18 Registries). Data were downloaded with SEER*Stat software (Version 8.3.9; The SEER Program, https://seer.cancer.gov). The inclusion criteria were listed as follows: (1) Age≥18 years old; (2) Diagnosed with pathological evidence; (3) Primary tumor and nodal status could be assessed; (4) Complete follow-up and known cause of death; (5) Surgery performed and known surgical approach. The stepwise extraction process from the SEER database is shown in **Figure 1**.

This study followed the Declaration of Helsinki (as revised in 2013). The SEER database is a public database without personal identifying information. Therefore, the ethical review was exempted, and no consent was needed.

### Definitions

Annual percentage change (APC) was utilized to describe trends of ratio. Demographic and clinical factors of patients were obtained from the SEER database. Continuous variables were converted to categorical variables based on the optimal cut-off values determined by the X-tile software (Version 3.6.1; Yale University, New Haven, CT, USA) or the Youden index (26). All patients were restaged to the current AJCC staging system (8^th^ edition). The regional lymph nodes were defined as the hilar, hepatoduodenal ligament, inferior phrenic, and caval lymph nodes. The clinical TNM stage (cTNM) was defined as the preoperative staging of HCC patients based on comprehensive evaluation and the AJCC staging system (8^th^ edition), which was approximated by the pathological stage in this study. Missing data and correlations among prognostic factors were considered in the analysis.

### Statistics

The Joinpoint Regression Program (Version 4.7.0; IMS; Calverton, MD, USA) was used to calculate the APC values (27). The cumulative incidences of cancer-specific death (CSD) and other cause-specific death (OCSD) were estimated using cumulative incidence function (CIF) curves and compared by Fine-Gray’s test. Categorical variables were shown as numbers and compared using the chi-square test or Fisher’s exact test or likelihood ratio test based on applicable conditions. One-to-one propensity score matching (PSM) was performed by the nearest-neighbor method within 0.20 standard deviations between the two groups. Due to the limited number of positive dependent variables, the least absolute shrinkage and selection operator (LASSO) regression algorithm was used to select candidate clinicopathological factors associated with LNM. Then independent risk factors were identified using multivariable binary logistic regression analysis *via* the ‘Forward: LR’ method.

Patients with LND were randomly divided into the training and validation set at a ratio of 1:1 for external validation. A nomogram was constructed based on independent risk factors to provide a visual tool for clinical use. The area under the receiver operating curve (AUROC), equal to Harrell’s C-index, was calculated to assess prognostic performance. Calibration curves were plotted by bootstrapping with 1000 resamples to evaluate the predictive accuracy. Decision curve analysis (DCA) and clinical impact curve (CIC) analysis were performed to estimate the clinical utility of the nomogram (28). A result was considered statistically significant when two-tailed P<0.05. All statistical analyses were completed using R (Version 3.6.3; The R Foundation for Statistical Computing, http://www.r-project.org) and SPSS (Version 26.0, IBM, Chicago, IL, USA).

## Results

### Baseline Characteristics and Survival Analyses of Patients With Hepatocellular Carcinoma

A total of 8,829 HCC patients were enrolled in this study. Based on the optimal cut-off values calculated by the X-tile software, the two continuous variables (Age and Tumor Size) were converted to categorical variables (**Figure S1**). The baseline characteristics and survival analyses of HCC patients are shown in **Table 1**. According to the AJCC staging system (8^th^ edition), most patients (86.7%) were categorized into the pT1-pT2 stage. Fifty-six (4.2%) of 1,346 (15.2%) patients with LND had LNM (pN1). Only a few patients (1.8%) were reported to have distant metastasis (pM1). Most patients with complete clinical data had well or moderately differentiated tumors (80.8%) and liver cirrhosis (68.4%).

**Table 1:** Baseline characteristics and competing risk survival analyses of HCC patients.

Factors	No. of Patients (N=8829)	Univariable		Multivariate	
		P-CSD	P-OCSD	SHR (95%CI)	*P*
Year of Diagnosis		<0.001	0.003		
2004-2009	4020 (45.5)			Reference	
2010-2015	4809 (54.5)			0.788 (0.736-0.845)	<0.001
Age		<0.001	<0.001		
≤72	7284 (82.5)			Reference	
>72	1545 (17.5)			1.206 (1.109-1.312)	<0.001
Gender		0.444	0.055		
Female	2219 (25.1)			Reference	
Male	6610 (74.9)			1.115 (1.029-1.207)	0.007
Race		<0.001	<0.001		
White	5752 (65.1)			Reference	
Asia-Pacific	1791 (20.3)			0.813 (0.745-0.888)	<0.001
Black	1154 (13.1)			1.041 (0.944-1.147)	0.420
Other	132 (1.5)			0.712 (0.526-0.965)	0.028
Income^†^		0.841	0.150		
Below the median	5109 (57.9)			Reference	
Above the median	3720 (42.1)			0.951 (0.887-1.019)	0.150
AFP		<0.001	<0.001		
Negative	2494 (28.2)			Reference	
Positive	4319 (48.9)			1.359 (1.252-1.476)	<0.001
Borderline/Unknown	2016 (22.8)			1.254 (1.140-1.380)	<0.001
First Malignant		0.030	<0.001		
Yes	7654 (84.7)			Reference	
No	1175 (13.3)			0.912 (0.824-1.009)	0.075
Neoadjuvant Therapy		<0.001	0.002		
Yes	992 (11.2)			Reference	
No	7837 (88.8)			1.164 (1.003-1.352)	0.046
Tumor Number		<0.001	0.052		
Single	6332 (71.7)			Reference	
Multiple	2497 (28.3)			1.054 (0.921-1.206)	0.440
Tumor Size		<0.001	<0.001		
≤28mm	3360 (38.1)			Reference	
28-95mm	4556 (51.6)			1.457 (1.325-1.603)	<0.001
>95mm	913 (10.3)			2.122 (1.845-2.440)	<0.001
Surgery		<0.001	<0.001		
LD	2510 (28.4)			Reference	
LR	3905 (44.2)			0.650 (0.598-0.708)	<0.001
LT	2414 (27.3)			0.227 (0.200-0.258)	<0.001
T Stage		<0.001	<0.001		
T1a	1250 (14.2)			Reference	
T1b	3821 (43.3)			0.907 (0.792-1.040)	0.160
T2	2583 (29.3)			1.157 (0.979-1.367)	0.086
T3	656 (7.4)			1.727 (1.365-2.185)	<0.001
T4	467 (5.3)			1.950 (1.602-2.373)	<0.001
TX	52 (0.6)			1.147 (0.765-1.720)	0.510
N Stage		<0.001	0.274		
N0	1290 (14.6)			Reference	
N1	56 (0.6)			1.785 (1.187-2.684)	0.005
NX	7483 (84.8)			1.053 (0.940-1.180)	0.370
M Stage		<0.001	0.350		
M0	8669 (98.2)			Reference	
M1	160 (1.8)			1.680 (1.323-2.134)	<0.001
Grade^‡^		<0.001	0.002		
G1-G2	5475 (62.0)			Reference	
G3-G4	1300 (14.7)			1.446 (1.311-1.595)	<0.001
Unknown	2054 (23.3)			1.141 (1.049-1.240)	0.002
Liver Cirrhosis		<0.001	0.276		
No	999 (11.3)			Reference	
Yes	2161 (24.5)			1.225 (1.079-1.390)	0.002
Unknown	5669 (64.2)			1.254 (1.140-1.380)	<0.001

HCC, Hepatocellular carcinoma; AFP, Alpha fetoprotein; LD, Local destruction; LR, Liver resection; LT, Liver transplantation; CSD, Cancer-specific death; OCSD, Other cause-specific death; SHR, Subdistribution hazard ratio; CI, Confidence interval.

^†^U.S. Census Bureau, Real Median Household Income in the United States [MEHOINUSA672N], retrieved from FRED, Federal Reserve Bank of St. Louis; https://fred.stlouisfed.org/series/MEHOINUSA672N, June 26, 2021.

^‡^G1, Well differentiated; G2, Moderately differentiated; G3, Poorly differentiated; G4, Undifferentiated.

The final follow-up was performed in November 2020, with a mean follow-up time of 56.0 ± 43.6 (IQR: 20.0, 83.0) months. During the follow-up period, 5,132 (58.1%) patients died, and 72.0% of deaths were attributable to HCC. The median cancer-specific survival time was 108.0 (95% CI=96.7-119.3) months. The 1year-, 3year- and 5year- cumulative incidences of CSD were 12.8%, 28.8% and 37.4%, respectively.

As shown in **Table 1**, **Table S1**; **Figure S2**, the CIF curves indicated that year of diagnosis, age, race, AFP, cancer history, neoadjuvant therapy, tumor size, surgery, T stage, and grade were likely to be associated with OCSD (all P<0.05). According to the multivariate competing risk analyses, LNM (pN1) was considered one of the independent prognostic factors of HCC patients (P=0.005). The 1year-, 3year- and 5year- cumulative incidences of CSD in patients with or without LNM were 39.3%, 69.6%, 78.6% and 9.8%, 19.9%, 26.5%, respectively. However, there was no survival difference between pN0 patients and those without LND (pNX, P=0.370).

### The Role of Regional Lymph Node Dissection in Patients With Hepatocellular Carcinoma

**Figure 2:**
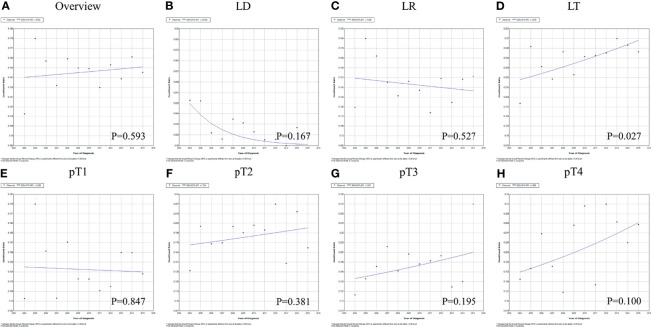
The variation trends and APC values for the rate of LND in HCC patients from 2004 to 2015: **(A)** all patients; **(B–D)** patients who underwent different surgeries; **(E–H)** patients with different tumor burdens. APC, Annual percent change; LND, Lymph node dissection; HCC, Hepatocellular carcinoma; LD, Local destruction; LR, Liver resection; LT, Liver transplantation.

In all, 1,346 (15.2%) HCC patients underwent LND. There were 2,497 lymph nodes retrieved from 1,287 patients with complete clinical data, and 93 (3.7%) of them were positive. The overall LND rate was 11.8% in 2004 and 15.1% in 2015, with an APC of 0.5% (95% CI=-1.5%-2.6%, P=0.593, **Figure 2A**). For different surgical approaches, the LND rate was 1.4%, 14.5% and 31.0% in patients who underwent local destruction (LD), liver resection (LR) and liver transplantation (LT), respectively (Chi-square=843.735, P<0.001). The trends of the LND rate in patients with LD and LR were similar to the overall cohort (P>0.05, **Figures 2B, C**). LND became increasingly common in patients with LT, and the APC was 3.3% (95% CI=0.5%-6.2%, P=0.027, **Figure 2D**). Considering different tumor burdens, the LND rates of pT1-pT2 and pT3-pT4 patients were 14.7% and 19.8% (Chi-square=19.490, P<0.001), and the LND rates remained stable between 2004 and 2015 (**Figures 2E–H**, all P>0.05).

**Figure 3:**
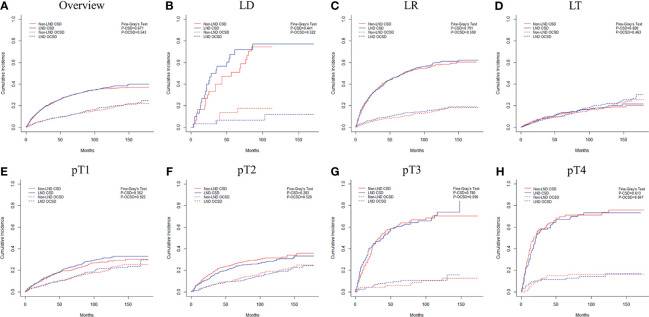
Cumulative incidence function curves of mortality of HCC patients with or without LND: **(A)** all patients; **(B–D)** patients who underwent different surgeries; **(E–H)** patients with different tumor burdens. HCC, Hepatocellular Carcinoma; LND, Lymph node dissection; CSD, Cancer-specific death; OCSD, Other cause-specific death; LD, Local destruction; LR, Liver resection; LT, Liver transplantation.

Data were further examined to explore the oncologic benefits of LND in HCC patients. After PSM (1,343 patients in each group), the LND group did not show better prognoses than the non-LND group (**Table S2** and **Figure 3A**, P=0.671). The same results were also obtained in subgroup analyses according to surgery (**Tables S3–S5** and **Figures 3B–D**, all P>0.05) and tumor burden (**Tables S6–S9** and **Figures 3E–H**, all P>0.05) after PSM. Another concern was whether increasing the number of lymph nodes retrieved could improve the outcomes of patients with LND. There were 748 (58.1%), 265 (20.6%), 139 (10.8%) and 135 (10.5%) patients who retrieved 1, 2, 3 and 4+ lymph nodes, respectively. As shown in **Table S10**, retrieving more lymph nodes showed no direct survival advantages (P>0.05). Therefore, the role of LND was to provide necessary staging information to identify patients with LNM. The ROC curve analysis confirmed that retrieving three or more lymph nodes showed the greatest discriminatory power of LNM (Youden index=0.211, sensitivity=0.415, specificity=0.796, **Figure S3A**).

### Development and Validation of a Model to Predict Regional Lymph Node Metastasis of Hepatocellular Carcinoma

**Figure 4:**
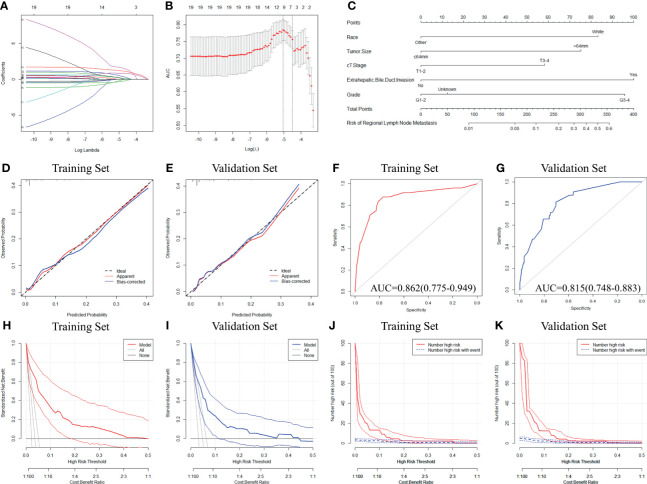
Development and validation of a novel model to predict LNM in HCC patients. **(A, B)** Variable selection process based on the LASSO logistic regression algorithm. **(C)** The nomogram to predict LNM in HCC patients was developed from the training set. **(D, E)** Calibration curve analyses of the constructed model in predicting LNM in the training and validation sets. **(F, G)** Receiver operating characteristic curve analyses of the constructed model in predicting LNM in the training and validation sets. **(H, I)** Decision curve analyses of the constructed model in predicting LNM in the training and validation sets. **(J, K)** Clinical impact curve analyses of the constructed model in predicting LNM in the training and validation sets. LNM, Lymph node metastasis; HCC, Hepatocellular carcinoma; LASSO, the least absolute shrinkage and selection operator.

Further analyses were performed in 1,346 patients with LND. Based on the optimal cut-off values determined by the Youden index, the two continuous variables (Age and Tumor Size) were converted to categorical variables (**Figures S3B, C**). The baseline characteristics of the patients in the training and validation sets are shown in **Table S11**. Seven factors with nonzero coefficients were filtered by the LASSO algorithm (**Figures 4A, B**), and the coefficients are listed in **Table S12**. According to the multiple logistic regression, white race, tumor size>64mm, cT3-4, G3-4, and extrahepatic bile duct invasion were considered independent risk factors for LNM in the training set (all P<0.05, **Table 2**).

**Table 2:** Multivariable analyses for preoperative risk factors for LNM in HCC patients.

Factors	No. of Patients (n=673)	OR (95%CI)	*P*
Gender			0.183
Female	181 (26.9)	–	
Male	492 (73.1)	–	
Race			
Other	205 (30.5)	Reference	
White	468 (69.5)	4.841 (1.480-15.836)	0.009
Tumor Size			
≤64mm	520 (77.3)	Reference	
>64mm	153 (22.7)	4.163 (1.485-11.669)	0.007
cT Stage			
cT1-2	562 (83.5)	Reference	
cT3-4	111 (16.5)	3.003 (1.121-8.045)	0.029
Major Vascular Invasion			0.594
No/Unknown	637 (94.7)	–	
Yes	36 (5.3)	–	
Extrahepatic Bile Duct Invasion^†^			
No/Unknown	662 (98.4)	Reference	
Yes	11 (1.6)	6.638 (1.019-43.239)	0.048
Grade^‡^			
G1-G2	431 (64.0)	Reference	
G3-G4	129 (19.2)	6.130 (2.293-16.386)	<0.001
Unknown	113 (16.8)	1.266 (0.249-6.434)	0.776

LNM, Lymph node metastasis; HCC, Hepatocellular carcinoma; OR, Odds ratio.

^†^Including gallbladder invasion.

^‡^G1, Well differentiated; G2=Moderately differentiated; G3, Poorly differentiated; G4, Undifferentiated.

The nomogram was constructed based on the independent risk factors to predict LNM in HCC patients (**Figure 4C**). The calibration curves showed good consistency between the predicted and observed probabilities in both the training and validation sets (**Figures 4D, E**). The AUROC values were 0.862 (95% CI=0.775-0.949) in the training set and 0.815 (95% CI=0.748-0.883) in the validation set (**Figures 4F, G**). To further estimate the clinical utility of the model, DCAs and CICs were introduced (**Figures 4H–K**). The nomogram provided a better net benefit than ‘all’ or ‘none’ schemes and showed a good clinical effect in both the training and validation sets. To further simplify the application of the nomogram, an online tool has been produced and published, which can be accessed through the following URL: https://chenxiaoyuan.shinyapps.io/HCC-RLNM/.

## Discussion

HCC is the most common subtype of primary liver cancer, with increasing morbidity, which was attributable to a higher incidence of viral hepatitis (especially HCV), nonalcoholic steatohepatitis, and metabolic syndrome (1, 5). Considering that these factors are all modifiable, the prevention of HCC still needs more effort. Over the past decades, HCC patients have benefitted from improved comprehensive therapies and surveillance programs. Nevertheless, more attention still should be given to developing better therapeutic strategies to further improve the prognosis of HCC.

Different from intrahepatic cholangiocarcinoma and combined hepatocellular-cholangiocarcinoma (CHC), LNM represents a rare but equally aggressive biological behavior of HCC, secondary only to the lung as the destination that most commonly develops extrahepatic metastasis (9, 29, 30). The incidence of LNM varies in different clinical observational studies, from 1.2% to 15.3% (10–17). Some possible confounding factors that could explain such a 10-fold variety were shown as follows: First, several researchers enrolled patients with fibrolamellar carcinoma even CHC, leading to an inappropriate increase in the overall LNM incidence. Second, enlarged lymph nodes might be not only metastases but also inflammatory responses, which could also cause false positives in diagnoses based on clinical experience. In this context, we set up a single subtype and pathologically confirmed cohort and investigated the actual LNM incidence to be 4.2%. During the long-term follow-up, many competing risks, such as comorbidities and accidents, may either hinder the observation or modify the chance of occurrence events of interest. The competing risk model could assess the informative nature of censoring and the occurrence rates of a particular event, so it is much more suitable than the standard Cox regression model in the present study. Multivariable survival analyses again confirmed that LNM was an independent predictor for HCC patients, which is not a surprising conclusion. Nevertheless, we found no OCSD difference in patients with different nodal statuses, indicating that LNM and LND could not worsen competing risk events. This finding indirectly proved the long-term safety of the LND procedure in HCC patients.

Currently, several rescue strategies to treat LNM in HCC patients have been proposed, including percutaneous ablation, intensity-modulated radiotherapy, and transcatheter arterial chemoembolization alone or in combination (31–35). However, the role of simultaneous LND during surgery is still a topic of debate. The overall LND rate was 15.2% in this study, which was consistent with previous studies (8.4%-20.1%) and showed no obvious fluctuation in the last decade (11, 15, 36). Specific to different surgical approaches, the LND rate was higher in LT recipients. It displayed an upward trend, which may be attributable to an increasingly standardized postoperative pathological examination of diseased liver. For patients with different tumor burdens, surgeons were more inclined to perform LND for those with locally advanced tumors. Overall, routine LND in HCC patients is not widely accepted by hepatobiliary surgeons in current clinical practice.

Some previous studies advocated routine LND for benefits in outcome improvement, complication prevention, and comprehensive evaluation, but some others hold the opposite opinions (10, 11, 23–25). Wu et al. conducted a prospective randomized controlled clinical trial and concluded that LND was safe but could not improve HCC patients’ prognoses. Although it is a high-grade evidence, all 116 lymph nodes retrieved from 41 patients were pathologically determined to be free of cancer, which may cause potential bias (23). Amini and colleagues were skeptical of routine LND in HCC patients after performing a systematic review, but the LND rate in 3 of 7 included studies was 100%, which could introduce publication bias (24). Under these circumstances, we again investigated the role of LND in a national real-world cohort to provide more evidence. After PSM, LND showed no oncologic benefit compared with the non-LND group. Subgroup analyses according to different surgical approaches and tumor burden also obtained the same finding. Further survival analyses confirmed that the increased number of lymph nodes retrieved could not improve prognoses. Therefore, the central role of LND in HCC patients was to offer staging information and identify patients with LNM for adjuvant therapy. To accurately evaluate nodal status, routine LND seems to be a suitable strategy in theory. LND has been confirmed not to worsen the prognoses and competing risks in this study and reported to be safe and well-tolerated in short-term outcomes (11, 14, 22, 23). Taking the rarity of LNM, prolonged surgery time, and low but not zero increased morbidity into account, however, performing LND for all patients may not be worthwhile.

Stratifying HCC patients with different risks of LNM and determining those who could benefit from LND is a challenging topic. The Liver Cancer Study Group of Japan reported that the incidence of LNM was 30.3% in 1374 autopsy cases, which significantly exceeded the data obtained from clinical practice. Considering that only 15.2% of patients received LND, a great number of patients without LND had occult LNM, indicating that the existing evaluation methods based on radiology are insufficient. Although previous studies have shown some factors that were associated with the presence of LNM, such as PET-CT imaging, dual-energy CT imaging, lncRNAs, microRNAs, and some other hematological indicators, these models were costly and required some technology to generate scores (12, 13, 15, 37–40). In the present study, with the help of the LASSO logistic regression algorithm, we incorporated five easily accessible factors, including race, tumor size, clinical T stage (cT stage), extrahepatic bile duct invasion, and tumor grade, to develop a model for predicting LNM in HCC patients. Tumor size, cT stage, and extrahepatic bile duct invasion could be evaluated *via* radiology imaging. The tumor grade data could be obtained from preoperative puncture (if applicable) or be marked as ‘Unknown’. We also provide a nomogram and a supporting online tool for individualized evaluation for further convenience of use. The model showed relatively high accuracy with AUROCs (or Harrell’s C-indexes) exceeding 0.800 and well-fitted calibration curves in both the training and validation sets. However, high prediction accuracy is not the same as a high clinical practical value. DCA and CIC could quantify the overall benefits of the prediction models based on the threshold probability introduced to this study to examine the value of the nomogram in clinical practice (28). The DCA and CIC demonstrated that the model had better clinical value than ‘none’ and ‘all’ and confirmed the validity of the model for predicting LNM in HCC patients.

In a population-based cohort, utilizing the SEER database, we investigated the prevalence of LNM, explored the role of LND, and developed and validated a novel model to stratify the risks of LNM in HCC patients. Although our study has many merits, including large sample size, complete follow-up, single pathological subtype, microscopically confirmed LNM, and competing risk survival analyses, there are still some limitations. First, the major drawback of this study is the inherent bias of the retrospective study. Second, the SEER database lacks detailed clinicopathological data, which caused unknown bias and limited further subgroup analysis. Nevertheless, only 56 patients had LNM even in our high-volume cohort due to the low incidence. Incorporating too many factors in the regression with limited positive dependent variables would lead to statistical instability and model overfitting and affect the reliability of the conclusions. Third, we could not identify patients with different liver function levels due to a lack of data, which was also considered a potential risk factor by several researchers (13, 22). It is evident that patients who underwent LR are more likely to have a better liver function. This study constructed a more representative cohort, including patients undergoing LR, LT, and LD, which could make up for this potential selection bias to a certain degree.

## Conclusion

LNM is an independent prognostic factor for HCC patients. Although LND shows no oncologic benefit, it could provide staging information to stratify patients and identify those who may have LNM. Nevertheless, routine LND in HCC patients is not necessary. Race, tumor size, clinical T stage, extrahepatic bile duct invasion, and tumor grade are independent risk factors for LNM. The constructed model could predict LNM with good performance, which is meaningful to optimize individualized treatment strategies in HCC patients.

## Data Availability Statement

Publicly available datasets were analyzed in this study. This data can be found here: https://seer.cancer.gov.

## Ethics Statement

Ethical review and approval was not required for the study on human participants in accordance with the local legislation and institutional requirements. Written informed consent for participation was not required for this study in accordance with the national legislation and the institutional requirements.

## Author Contributions

XW is the lead contact for this article. XW, YG, and XC conceived and designed the study. XW and YG supervised the study and offered administrative support. YL, XS, and JZ collected and assembled the data. XC, YL, and GH analyzed and interpreted the data. XC and XS wrote the manuscript. XW and YG reviewed the manuscript. All the authors read and finally approved the manuscript.

## Funding

This study was supported by grants from the National Natural Science Foundation of China (Grant No. 31930020, 81870488, 81521004, 81530048), Natural Science Foundation of Jiangsu Province (Grant No. BK20170142) and Key Laboratory of Liver Transplantation, Chinese Academy of Medical Sciences (Grant No. 2018PT31043, 2019PT320015).

## Conflict of Interest

The authors declare that the research was conducted in the absence of any commercial or financial relationships that could be construed as a potential conflict of interest.

## Publisher’s Note

All claims expressed in this article are solely those of the authors and do not necessarily represent those of their affiliated organizations, or those of the publisher, the editors and the reviewers. Any product that may be evaluated in this article, or claim that may be made by its manufacturer, is not guaranteed or endorsed by the publisher.
