# Supplementary material for: Development and Validation of a Novel Model to Predict Regional Lymph Node Metastasis in Patients With Hepatocellular Carcinoma
Source: Front Oncol. 2022 Feb 11;12:835957. doi: 10.3389/fonc.2022.835957 (PMC8874317; doi:10.3389/fonc.2022.835957)
Supplement: Supplementary file 1 [file Image_1.pdf]

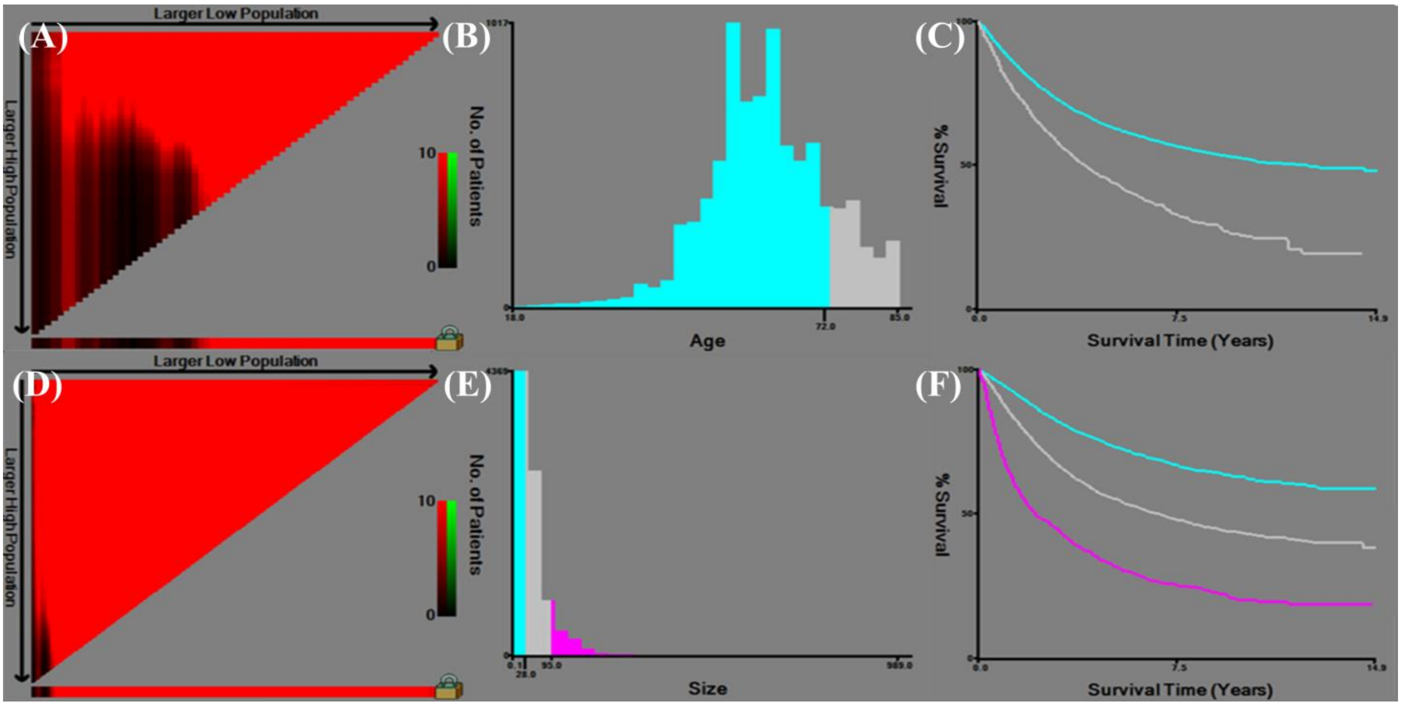

Figure S1 X-tile plots for determining optimal cut-off values of **(A)-(C)** age and **(D)-(F)** tumor size according to cancer-specific survival.
