# Supplementary material for: Development and Validation of a Novel Model to Predict Regional Lymph Node Metastasis in Patients With Hepatocellular Carcinoma
Source: Front Oncol. 2022 Feb 11;12:835957. doi: 10.3389/fonc.2022.835957 (PMC8874317; doi:10.3389/fonc.2022.835957)
Supplement: Supplementary file 2 [file Image_2.pdf]

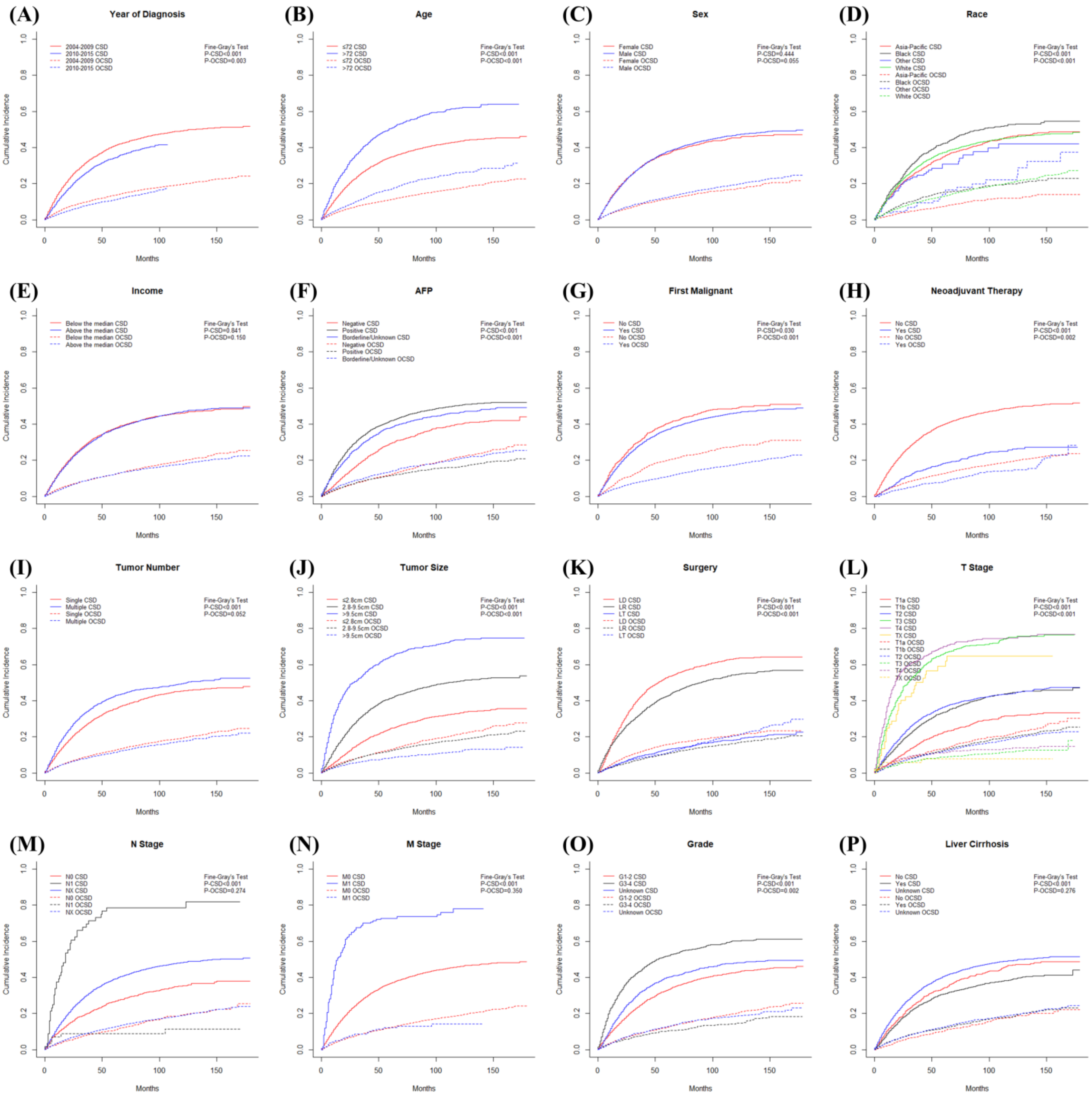

Figure S2 Cumulative incidence functions of mortality of HCC patients according to **(A)** Year of Diagnosis, **(B)** Age, **(C)** Sex, **(D)** Race, **(E)** Income, **(F)** AFP, **(G)** First Malignant, **(H)** Neoadjuvant Therapy, **(I)** Tumor Number, **(J)** Tumor Size, **(K)** Surgery, **(L)** pT stage, **(M)** pN stage, **(N)** pM stage, **(O)** Tumor Grade, **(P)** Liver Cirrhosis.

HCC, Hepatocellular carcinoma; AFP, Alpha fetoprotein; LD, Local destruction; LR, Liver resection; LT, Liver transplantation; CSD, Cancer-specific death; OCSD, Other cause-specific death.
