# Supplementary material for: Development and Validation of a Novel Model to Predict Regional Lymph Node Metastasis in Patients With Hepatocellular Carcinoma
Source: Front Oncol. 2022 Feb 11;12:835957. doi: 10.3389/fonc.2022.835957 (PMC8874317; doi:10.3389/fonc.2022.835957)
Supplement: Supplementary file 3 [file Image_3.pdf]

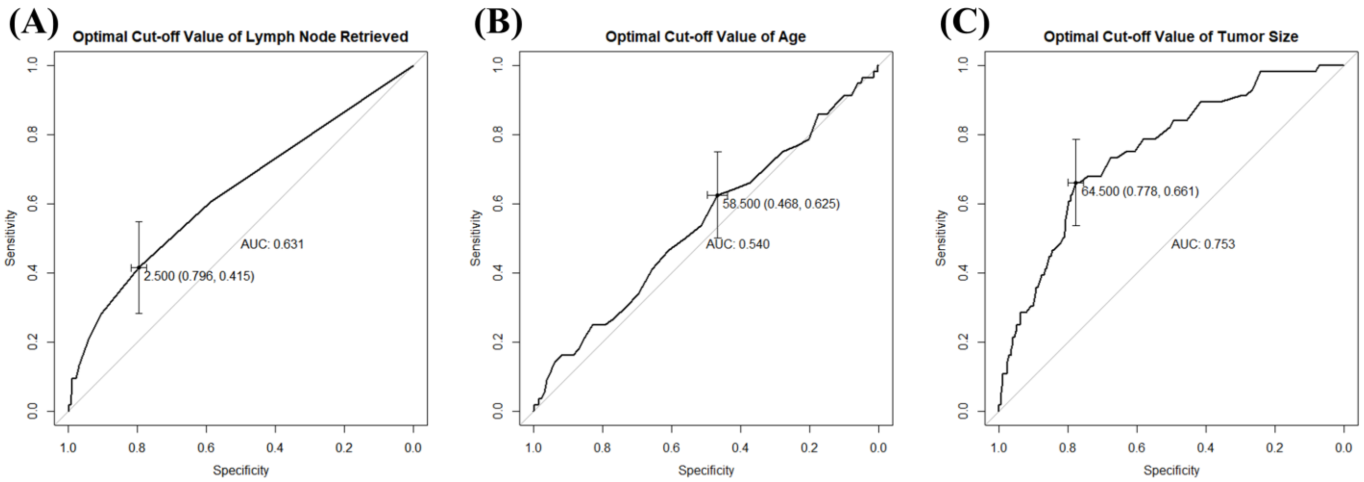

Figure S3 Receiver operating characteristic curve analyses for determining optimal cut-off values of **(A)** number of lymph nodes retrieved, **(B)** age, **(C)** tumor size based on Youden index.
