# Supplementary material for: Development and Validation of a Novel Model to Predict Regional Lymph Node Metastasis in Patients With Hepatocellular Carcinoma
Source: Front Oncol. 2022 Feb 11;12:835957. doi: 10.3389/fonc.2022.835957 (PMC8874317; doi:10.3389/fonc.2022.835957)
Supplement: Supplementary file 4 [file Table_1.docx]

**Supplementary Tables**

| Contents | | |
| --- | --- | --- |
| Table | Legends | Page |
| Table S1 | Cumulative incidence of CSD and OCSD of HCC patients | 2 |
| Table S2 | Comparison between LND and Non-LND before and after PSM in all HCC patients | 4 |
| Table S3 | Comparison between LND and Non-LND before and after PSM in HCC patients with LD | 6 |
| Table S4 | Comparison between LND and Non-LND before and after PSM in HCC patients with LR | 8 |
| Table S5 | Comparison between LND and Non-LND before and after PSM in HCC patients with LT | 10 |
| Table S6 | Comparison between LND and Non-LND before and after PSM in pT1 HCC patients | 12 |
| Table S7 | Comparison between LND and Non-LND before and after PSM in pT2 HCC patients | 14 |
| Table S8 | Comparison between LND and Non-LND before and after PSM in pT3 HCC patients | 16 |
| Table S9 | Comparison between LND and Non-LND before and after PSM in pT4 HCC patients | 18 |
| Table S10 | Competing risk survival analyses of HCC patients with LND | 20 |
| Table S11 | Baseline characteristics of HCC patients with LND in the training and validation set | 22 |
| Table S12 | The coefficient for each factor in the LASSO regression models | 24 |

Table S1. Cumulative incidence of CSD and OCSD of HCC patients

| Factors | CSD | | | OCSD | | |
| --- | --- | --- | --- | --- | --- | --- |
|  | 1yr-CI | 3yr-CI | 5yr-CI | 1yr-CI | 3yr-CI | 5yr-CI |
| Year of Diagnosis |  |  |  |  |  |  |
| 2004-2009 | 0.149 | 0.319 | 0.409 | 0.048 | 0.100 | 0.136 |
| 2010-2015 | 0.111 | 0.262 | 0.341 | 0.033 | 0.079 | 0.109 |
| Age |  |  |  |  |  |  |
| ≤72 | 0.114 | 0.264 | 0.346 | 0.038 | 0.083 | 0.112 |
| >72 | 0.195 | 0.402 | 0.506 | 0.050 | 0.115 | 0.169 |
| Gender |  |  |  |  |  |  |
| Female | 0.134 | 0.288 | 0.364 | 0.038 | 0.084 | 0.116 |
| Male | 0.126 | 0.288 | 0.377 | 0.041 | 0.090 | 0.124 |
| Race |  |  |  |  |  |  |
| White | 0.128 | 0.289 | 0.373 | 0.044 | 0.096 | 0.130 |
| Asia-Pacific | 0.120 | 0.264 | 0.349 | 0.022 | 0.051 | 0.074 |
| Black | 0.142 | 0.327 | 0.422 | 0.048 | 0.108 | 0.153 |
| Other | 0.115 | 0.238 | 0.308 | 0.038 | 0.077 | 0.139 |
| Income^†^ |  |  |  |  |  |  |
| Below the median | 0.129 | 0.291 | 0.374 | 0.043 | 0.089 | 0.122 |
| Above the median | 0.127 | 0.284 | 0.373 | 0.035 | 0.087 | 0.122 |
| AFP |  |  |  |  |  |  |
| Negative | 0.069 | 0.200 | 0.293 | 0.036 | 0.084 | 0.122 |
| Positive | 0.158 | 0.335 | 0.417 | 0.039 | 0.086 | 0.115 |
| Borderline/Unknown | 0.137 | 0.297 | 0.381 | 0.047 | 0.099 | 0.138 |
| First Malignant |  |  |  |  |  |  |
| Yes | 0.151 | 0.306 | 0.402 | 0.057 | 0.138 | 0.198 |
| No | 0.125 | 0.285 | 0.369 | 0.037 | 0.081 | 0.111 |
| Neoadjuvant Therapy |  |  |  |  |  |  |
| Yes | 0.045 | 0.137 | 0.183 | 0.022 | 0.059 | 0.084 |
| No | 0.139 | 0.307 | 0.398 | 0.042 | 0.092 | 0.127 |
| Tumor Number |  |  |  |  |  |  |
| Single | 0.116 | 0.268 | 0.354 | 0.040 | 0.091 | 0.125 |
| Multiple | 0.159 | 0.339 | 0.424 | 0.039 | 0.083 | 0.114 |
| Tumor Size |  |  |  |  |  |  |
| ≤28mm | 0.063 | 0.175 | 0.246 | 0.044 | 0.092 | 0.131 |
| 28-95mm | 0.135 | 0.321 | 0.414 | 0.038 | 0.090 | 0.124 |
| >95mm | 0.337 | 0.544 | 0.645 | 0.033 | 0.065 | 0.083 |
| Surgery |  |  |  |  |  |  |
| LD | 0.168 | 0.418 | 0.529 | 0.051 | 0.115 | 0.157 |
| LR | 0.161 | 0.325 | 0.427 | 0.033 | 0.074 | 0.107 |
| LT | 0.034 | 0.094 | 0.130 | 0.039 | 0.083 | 0.112 |
| T Stage |  |  |  |  |  |  |
| T1a | 0.050 | 0.153 | 0.227 | 0.050 | 0.097 | 0.136 |
| T1b | 0.098 | 0.248 | 0.335 | 0.036 | 0.090 | 0.127 |
| T2 | 0.118 | 0.282 | 0.365 | 0.039 | 0.087 | 0.120 |
| T3 | 0.296 | 0.552 | 0.660 | 0.032 | 0.066 | 0.090 |
| T4 | 0.388 | 0.619 | 0.702 | 0.056 | 0.100 | 0.111 |
| TX | 0.250 | 0.504 | 0.592 | 0.058 | 0.058 | 0.079 |
| N Stage |  |  |  |  |  |  |
| N0 | 0.098 | 0.199 | 0.265 | 0.036 | 0.081 | 0.106 |
| N1 | 0.393 | 0.696 | 0.786 | 0.071 | 0.089 | 0.089 |
| NX | 0.131 | 0.300 | 0.389 | 0.041 | 0.090 | 0.125 |
| M Stage |  |  |  |  |  |  |
| M0 | 0.122 | 0.280 | 0.367 | 0.040 | 0.088 | 0.122 |
| M1 | 0.469 | 0.700 | 0.728 | 0.050 | 0.088 | 0.122 |
| Grade^‡^ |  |  |  |  |  |  |
| G1-G2 | 0.102 | 0.244 | 0.330 | 0.040 | 0.090 | 0.125 |
| G3-G4 | 0.237 | 0.434 | 0.519 | 0.040 | 0.076 | 0.104 |
| Unknown | 0.129 | 0.315 | 0.400 | 0.038 | 0.092 | 0.127 |
| Liver Cirrhosis |  |  |  |  |  |  |
| No | 0.113 | 0.259 | 0.341 | 0.024 | 0.069 | 0.103 |
| Yes | 0.096 | 0.234 | 0.308 | 0.044 | 0.092 | 0.121 |
| Unknown | 0.143 | 0.314 | 0.405 | 0.041 | 0.090 | 0.126 |

CSD=Cancer-specific death; OCSD=Other cause-specific death; HCC=Hepatocellular carcinoma; CI=Confidence interval; AFP=Alpha fetoprotein; LD=Local destruction; LR=Liver resection; LT=Liver transplantation.

^†^U.S. Census Bureau, Real Median Household Income in the United States [MEHOINUSA672N], retrieved from FRED, Federal Reserve Bank of St. Louis; https://fred.stlouisfed.org/series/MEHOINUSA672N, June 26, 2021

^‡^G1=Well differentiated; G2=Moderately differentiated; G3=Poorly differentiated; G4=Undifferentiated.

Table S2. Comparison between LND and Non-LND before and after PSM in all HCC patients

| Factors | Before PSM | | | After PSM | | |
| --- | --- | --- | --- | --- | --- | --- |
|  | Non-LND  (n=7483) | LND  (n=1346) | P | Non-LND  (n=1343) | LND  (n=1343) | P |
| Year of Diagnosis |  |  | 0.922 |  |  | 0.699 |
| 2004-2009 | 3405(45.5) | 615(45.7) |  | 624(46.5) | 613(45.6) |  |
| 2010-2015 | 4078(54.5) | 731(54.3) |  | 719(53.5) | 730(54.4) |  |
| Age |  |  | <0.001 |  |  | 0.571 |
| ≤72 | 6051(80.9) | 1233(91.6) |  | 1239(92.3) | 1230(91.6) |  |
| >72 | 1432(19.1) | 113(8.4) |  | 104(7.7) | 113(8.4) |  |
| Gender |  |  | 0.672 |  |  | 0.792 |
| Female | 1874(25.0) | 345(25.6) |  | 351(26.1) | 344(25.6) |  |
| Male | 5609(75.0) | 1001(74.4) |  | 992(73.9) | 999(74.4) |  |
| Race |  |  | <0.001 |  |  | 0.846 |
| White | 4812(64.3) | 940(69.8) |  | 960(71.5) | 939(69.9) |  |
| Asia-Pacific | 1594(21.3) | 197(14.6) |  | 187(13.9) | 197(14.7) |  |
| Black | 958(12.8) | 196(14.6) |  | 183(13.6) | 194(14.4) |  |
| Other | 119(1.6) | 13(1.0) |  | 13(1.0) | 13(1.0) |  |
| Income^†^ |  |  | 0.050 |  |  | 0.665 |
| Below the median | 4297(57.4) | 812(60.3) |  | 799(59.5) | 811(60.4) |  |
| Above the median | 3186(42.6) | 534(39.7) |  | 544(40.5) | 532(39.6) |  |
| AFP |  |  | 0.012 |  |  | 0.289 |
| Negative | 2153(28.8) | 341(25.3) |  | 374(27.8) | 341(25.4) |  |
| Positive | 3614(48.3) | 705(52.4) |  | 667(49.7) | 703(52.3) |  |
| Borderline/Unknown | 1716(22.9) | 300(22.3) |  | 302(22.5) | 299(22.3) |  |
| First Malignant |  |  | <0.001 |  |  | 0.127 |
| Yes | 6446(86.1) | 1208(89.7) |  | 1230(91.6) | 1206(89.8) |  |
| No | 1037(13.9) | 138(10.3) |  | 113(8.4) | 137(10.2) |  |
| Neoadjuvant Therapy |  |  | <0.001 |  |  | 0.860 |
| Yes | 643(8.6) | 349(25.9) |  | 341(25.4) | 346(25.8) |  |
| No | 6840(91.4) | 997(74.1) |  | 1002(74.6) | 997(74.2) |  |
| Tumor Number |  |  | <0.001 |  |  | 0.716 |
| Single | 5465(73.0) | 867(64.4) |  | 876(65.2) | 866(64.5) |  |
| Multiple | 2018(27.0) | 479(35.6) |  | 467(34.8) | 477(35.5) |  |
| Tumor Size |  |  | <0.001 |  |  | 0.770 |
| ≤28mm | 2829(37.8) | 531(39.5) |  | 527(39.2) | 531(39.5) |  |
| 28-95mm | 3930(52.5) | 626(46.5) |  | 638(47.5) | 623(46.4) |  |
| >95mm | 724(9.7) | 189(14.0) |  | 178(13.3) | 189(14.1) |  |
| Surgery |  |  | <0.001 |  |  | 0.911 |
| LD | 2478(33.1) | 32(2.4) |  | 31(2.3) | 32(2.4) |  |
| LR | 3339(44.6) | 566(42.1) |  | 556 (41.4) | 566 (42.1) |  |
| LT | 1666(22.3) | 748(55.6) |  | 756 (56.3) | 745 (55.5) |  |
| T Stage |  |  | <0.001 |  |  | 0.969 |
| T1a | 1069(14.3) | 181 (13.4) |  | 190(14.1) | 181(13.5) |  |
| T1b | 3359(44.9) | 462 (34.3) |  | 456(34.0) | 462(34.4) |  |
| T2 | 2102(28.1) | 481 (35.7) |  | 488(36.3) | 481(35.8) |  |
| T3 | 536(7.2) | 120(8.9) |  | 114(8.5) | 120(8.9) |  |
| T4 | 365(4.9) | 102(7.6) |  | 95(7.1) | 99(7.4) |  |
| TX | 52(0.7) | 0(0) |  | 0(0) | 0(0) |  |
| M Stage |  |  | 1.000 |  |  | 1.000 |
| M0 | 7347(98.2) | 1322(98.2) |  | 1319(98.2) | 1319(98.2) |  |
| M1 | 136(1.8) | 24 (1.8) |  | 24(1.8) | 24(1.8) |  |
| Grade^‡^ |  |  | <0.001 |  |  | 0.451 |
| G1-G2 | 4600(61.5) | 875(65.0) |  | 883(65.7) | 874(65.1) |  |
| G3-G4 | 1049(14.0) | 251(18.6) |  | 226(16.8) | 249(18.5) |  |
| Unknown | 1834(24.5) | 220(16.3) |  | 234(17.4) | 220(16.4) |  |
| Liver Cirrhosis |  |  | <0.001 |  |  | 0.278 |
| No | 824(11.0) | 175(13.0) |  | 151(11.2) | 175(13.0) |  |
| Yes | 1745(23.3) | 416(30.9) |  | 441(32.8) | 415(30.9) |  |
| Unknown | 4914(65.7) | 755(56.1) |  | 751(55.9) | 753(56.1) |  |
| 5yr-CSD | 0.389 | 0.287 | <0.001 | 0.286 | 0.287 | 0.671 |
| 5yr-OCSD | 0.125 | 0.106 | 0.450 | 0.106 | 0.106 | 0.543 |

LND=Lymph node dissection; PSM=Propensity score matching; HCC=Hepatocellular carcinoma; AFP=Alpha fetoprotein; LD=Local destruction; LR=Liver resection; LT=Liver transplantation; CSD=Cancer-specific death; OCSD=Other cause-specific death.

^†^U.S. Census Bureau, Real Median Household Income in the United States [MEHOINUSA672N], retrieved from FRED, Federal Reserve Bank of St. Louis; https://fred.stlouisfed.org/series/MEHOINUSA672N, June 26, 2021

^‡^G1=Well differentiated; G2=Moderately differentiated; G3=Poorly differentiated; G4=Undifferentiated.

Table S3. Comparison between LND and Non-LND before and after PSM in HCC patients with LD

| Factors | Before PSM | | | After PSM | | |
| --- | --- | --- | --- | --- | --- | --- |
|  | Non-LND  (n=2478) | LND  (n=32) | P | Non-LND  (n=30) | LND  (n=30) | P |
| Year of Diagnosis |  |  | 0.005 |  |  | 0.770 |
| 2004-2009 | 1131(45.6) | 23(71.9) |  | 23(76.7) | 21(70.0) |  |
| 2010-2015 | 1347(54.4) | 9 (28.1) |  | 7(23.3) | 9(30.0) |  |
| Age |  |  | 0.142 |  |  | 0.667 |
| ≤72 | 1848(74.6) | 28(87.5) |  | 28(93.3) | 26(86.7) |  |
| >72 | 630(25.4) | 4(12.5) |  | 2(6.7) | 4(13.3) |  |
| Gender |  |  | 0.621 |  |  | 0.729 |
| Female | 597(24.1) | 597(24.1) |  | 4 (13.3) | 6 (20.0) |  |
| Male | 1881(75.9) | 26(81.2) |  | 26 (86.7) | 24 (80.0) |  |
| Race |  |  | 0.078 |  |  | 0.839 |
| White | 1629(65.7) | 28(87.5) |  | 27(90.0) | 26(86.7) |  |
| Asia-Pacific | 462(18.6) | 2(6.2) |  | 1(3.3) | 2(6.7) |  |
| Black | 336(13.6) | 2(6.2) |  | 2(6.7) | 2(6.7) |  |
| Other | 51(2.1) | 0(0) |  | 0(0) | 0(0) |  |
| Income^†^ |  |  | 0.045 |  |  | 1.000 |
| Below the median | 1331(53.7) | 11(34.4) |  | 11(36.7) | 10(33.3) |  |
| Above the median | 1147(46.3) | 21(65.6) |  | 19(63.3) | 20(66.7) |  |
| AFP |  |  | 0.325 |  |  | 0.404 |
| Negative | 669(27.0) | 5(15.6) |  | 2(6.7) | 5(16.7) |  |
| Positive | 1332(53.8) | 19(59.4) |  | 18(60.0) | 18(60.0) |  |
| Borderline/Unknown | 477(19.2) | 8(25.0) |  | 10(33.3) | 7(23.3) |  |
| First Malignant |  |  | 0.992 |  |  | 1.000 |
| Yes | 2050(82.7) | 27(84.4) |  | 26(86.7) | 25(83.3) |  |
| No | 428(17.3) | 5(15.6) |  | 4(13.3) | 5(16.7) |  |
| Neoadjuvant Therapy |  |  | <0.001 |  |  | 1.000 |
| Yes | 15(0.6) | 3(9.4) |  | 0(0) | 1(3.3) |  |
| No | 2463(99.4) | 29(90.6) |  | 30(100.0) | 29(96.7) |  |
| Tumor Number |  |  | 1.000 |  |  | 0.778 |
| Single | 1752(70.7) | 23(71.9) |  | 20(66.7) | 22(73.3) |  |
| Multiple | 726(29.3) | 9(28.1) |  | 10(33.3) | 8(26.7) |  |
| Tumor Size |  |  | 0.292 |  |  | 1.000 |
| ≤28mm | 1064(42.9) | 12(37.5) |  | 9(30.0) | 10(33.3) |  |
| 28-95mm | 1291(52.1) | 20(62.5) |  | 21(70.0) | 20(66.7) |  |
| >95mm | 123(5.0) | 0(0) |  | 0(0) | 0(0) |  |
| T Stage |  |  | 0.675 |  |  | 0.945 |
| T1a | 372(15.0) | 3(9.4) |  | 2(6.7) | 2(6.7) |  |
| T1b | 1187(47.9) | 15(46.9) |  | 16(53.3) | 15(50.0) |  |
| T2 | 641(25.9) | 11(34.4) |  | 9(30.0) | 10(33.3) |  |
| T3 | 152(6.1) | 1(3.1) |  | 2(6.7) | 1(3.3) |  |
| T4 | 88(3.6) | 2(6.2) |  | 1(3.3) | 2(6.7) |  |
| TX | 38(1.5) | 0(0) |  | 0(0) | 0(0) |  |
| M Stage |  |  | 0.739 |  |  | - |
| M0 | 2416(97.5) | 32(100.0) |  | 30(100.0) | 30(100.0) |  |
| M1 | 62(2.5) | 0(0) |  | 0(0) | 0(0) |  |
| Grade^‡^ |  |  | 0.954 |  |  | 0.238 |
| G1-G2 | 1338(54.0) | 18(56.2) |  | 19(63.3) | 17(56.7) |  |
| G3-G4 | 183(7.4) | 2(6.2) |  | 5(16.7) | 2(6.7) |  |
| Unknown | 957(38.6) | 12(37.5) |  | 6(20.0) | 11(36.7) |  |
| Liver Cirrhosis |  |  | 0.665 |  |  | 0.706 |
| No | 157(6.3) | 3(9.4) |  | 4(13.3) | 3(10.0) |  |
| Yes | 590(23.8) | 6(18.8) |  | 7(23.3) | 5(16.7) |  |
| Unknown | 1731(69.9) | 23(71.9) |  | 19(63.3) | 22(73.3) |  |
| 5yr-CSD | 0.527 | 0.633 | 0.165 | 0.510 | 0.677 | 0.441 |
| 5yr-OCSD | 0.158 | 0.063 | 0.135 | 0.138 | 0.067 | 0.522 |

LND=Lymph node dissection; PSM=Propensity score matching; HCC=Hepatocellular carcinoma; LD=Local destruction; AFP=Alpha fetoprotein; CSD=Cancer-specific death; OCSD=Other cause-specific death.

^†^U.S. Census Bureau, Real Median Household Income in the United States [MEHOINUSA672N], retrieved from FRED, Federal Reserve Bank of St. Louis; https://fred.stlouisfed.org/series/MEHOINUSA672N, June 26, 2021

^‡^G1=Well differentiated; G2=Moderately differentiated; G3=Poorly differentiated; G4=Undifferentiated.

Table S4. Comparison between LND and Non-LND before and after PSM in HCC patients with LR

| Factors | Before PSM | | | After PSM | | |
| --- | --- | --- | --- | --- | --- | --- |
|  | Non-LND  (n=3339) | LND  (n=566) | P | Non-LND  (n=566) | LND  (n=566) | P |
| Year of Diagnosis |  |  | 0.397 |  |  | 0.952 |
| 2004-2009 | 1402(42.0) | 249(44.0) |  | 251(44.3) | 249(44.0) |  |
| 2010-2015 | 1937(58.0) | 317(56.0) |  | 315(55.7) | 317(56.0) |  |
| Age |  |  | 0.018 |  |  | 0.210 |
| ≤72 | 2554(76.5) | 459(81.1) |  | 476(84.1) | 459(81.1) |  |
| >72 | 785(23.5) | 107(18.9) |  | 90(15.9) | 107(18.9) |  |
| Gender |  |  | 0.170 |  |  | 0.948 |
| Female | 912(27.3) | 171(30.2) |  | 173(30.6) | 171(30.2) |  |
| Male | 2427(72.7) | 395(69.8) |  | 393(69.4) | 395(69.8) |  |
| Race |  |  | 0.020 |  |  | 0.436 |
| White | 1903(57.0) | 321(56.7) |  | 346(61.1) | 321(56.7) |  |
| Asia-Pacific | 916(27.4) | 133(23.5) |  | 125(22.1) | 133(23.5) |  |
| Black | 474(14.2) | 106(18.7) |  | 91(16.1) | 106(18.7) |  |
| Other | 46(1.4) | 6(1.1) |  | 4(0.7) | 6(1.1) |  |
| Income^†^ |  |  | 0.821 |  |  | 0.904 |
| Below the median | 1885(56.5) | 323(57.1) |  | 326(57.6) | 323(57.1) |  |
| Above the median | 1454(43.5) | 243(42.9) |  | 240(42.4) | 243(42.9) |  |
| AFP |  |  | 0.041 |  |  | 0.873 |
| Negative | 961(28.8) | 145(25.6) |  | 152(26.9) | 145(25.6) |  |
| Positive | 1531(45.9) | 292(51.6) |  | 290(51.2) | 292(51.6) |  |
| Borderline/Unknown | 847(25.4) | 129(22.8) |  | 124(21.9) | 129(22.8) |  |
| First Malignant |  |  | 0.376 |  |  | 0.289 |
| Yes | 2815(84.3) | 486(85.9) |  | 499(88.2) | 486(85.9) |  |
| No | 524(15.7) | 80(14.1) |  | 67(11.8) | 80(14.1) |  |
| Neoadjuvant Therapy |  |  | 0.020 |  |  | 0.537 |
| Yes | 146(4.4) | 38(6.7) |  | 32(5.7) | 38(6.7) |  |
| No | 3193(95.6) | 528(93.3) |  | 534(94.3) | 528(93.3) |  |
| Tumor Number |  |  | 0.002 |  |  | 0.892 |
| Single | 2675(80.1) | 420(74.2) |  | 417(73.7) | 420(74.2) |  |
| Multiple | 664(19.9) | 146(25.8) |  | 149(26.3) | 146(25.8) |  |
| Tumor Size |  |  | <0.001 |  |  | 0.788 |
| ≤28mm | 733(22.0) | 72(12.7) |  | 71(12.5) | 72(12.7) |  |
| 28-95mm | 2015(60.3) | 307(54.2) |  | 318(56.2) | 307(54.2) |  |
| >95mm | 591(17.7) | 187(33.0) |  | 177(31.3) | 187(33.0) |  |
| T Stage |  |  | <0.001 |  |  | 0.944 |
| T1a | 288(8.6) | 26(4.6) |  | 31(5.5) | 26(4.6) |  |
| T1b | 1648(49.4) | 225(39.8) |  | 222(39.2) | 225(39.8) |  |
| T2 | 798(23.9) | 137(24.2) |  | 135(23.9) | 137(24.2) |  |
| T3 | 345(10.3) | 95(16.8) |  | 100(17.7) | 95(16.8) |  |
| T4 | 248(7.4) | 83(14.7) |  | 78(13.8) | 83(14.7) |  |
| TX | 12(0.4) | 0(0) |  | 0(0) | 0(0) |  |
| M Stage |  |  | 0.062 |  |  | 0.872 |
| M0 | 3272(98.0) | 547(96.6) |  | 545(96.3) | 547(96.6) |  |
| M1 | 67(2.0) | 19(3.4) |  | 21(3.7) | 19(3.4) |  |
| Grade^‡^ |  |  | <0.001 |  |  | 0.712 |
| G1-G2 | 2231(66.8) | 354(62.5) |  | 357(63.1) | 354(62.5) |  |
| G3-G4 | 731(21.9) | 169(29.9) |  | 173(30.6) | 169(29.9) |  |
| Unknown | 377(11.3) | 43(7.6) |  | 36(6.4) | 43(7.6) |  |
| Liver Cirrhosis |  |  | 0.211 |  |  | 0.639 |
| No | 594(17.8) | 118(20.8) |  | 121(21.4) | 118(20.8) |  |
| Yes | 530(15.9) | 84(14.8) |  | 73(12.9) | 84(14.8) |  |
| Unknown | 2215(66.3) | 364(64.3) |  | 372(65.7) | 364(64.3) |  |
| 5yr-CSD | 0.420 | 0.466 | 0.002 | 0.470 | 0.466 | 0.791 |
| 5yr-OCSD | 0.105 | 0.116 | 0.818 | 0.092 | 0.116 | 0.590 |

LND=Lymph node dissection; PSM=Propensity score matching; HCC=Hepatocellular carcinoma; LR=Liver Resection; AFP=Alpha fetoprotein; CSD=Cancer-specific death; OCSD=Other cause-specific death.

^†^U.S. Census Bureau, Real Median Household Income in the United States [MEHOINUSA672N], retrieved from FRED, Federal Reserve Bank of St. Louis; https://fred.stlouisfed.org/series/MEHOINUSA672N, June 26, 2021

^‡^G1=Well differentiated; G2=Moderately differentiated; G3=Poorly differentiated; G4=Undifferentiated.

Table S5. Comparison between LND and Non-LND before and after PSM in HCC patients with LT

| Factors | Before PSM | | | After PSM | | |
| --- | --- | --- | --- | --- | --- | --- |
|  | Non-LND  (n=1666) | LND  (n=748) | P | Non-LND  (n=745) | LND  (n=745) | P |
| Year of Diagnosis |  |  | 0.004 |  |  | 0.835 |
| 2004-2009 | 872(52.3) | 343(45.9) |  | 337(45.2) | 342(45.9) |  |
| 2010-2015 | 794(47.7) | 405(54.1) |  | 408(54.8) | 403(54.1) |  |
| Age |  |  | 0.092 |  |  | 1.000 |
| ≤72 | 1649(99.0) | 746(99.7) |  | 744(99.9) | 743(99.7) |  |
| >72 | 17(1.0) | 2(0.3) |  | 1(0.1) | 2(0.3) |  |
| Gender |  |  | 0.803 |  |  | 0.530 |
| Female | 365(21.9) | 168(22.5) |  | 157(21.1) | 168(22.6) |  |
| Male | 1301(78.1) | 580(77.5) |  | 588(78.9) | 577(77.4) |  |
| Race |  |  | 0.002 |  |  | 0.677 |
| White | 1280(76.8) | 591(79.0) |  | 608(81.6) | 591(79.3) |  |
| Asia-Pacific | 216(13.0) | 62(8.3) |  | 56(7.5) | 62(8.3) |  |
| Black | 148(8.9) | 88(11.8) |  | 73(9.8) | 85(11.4) |  |
| Other | 22(1.3) | 7(0.9) |  | 8(1.1) | 7(0.9) |  |
| Income^†^ |  |  | 0.674 |  |  | 0.957 |
| Below the median | 1081(64.9) | 478(63.9) |  | 475(63.8) | 477(64.0) |  |
| Above the median | 585(35.1) | 270(36.1) |  | 270(36.2) | 268(36.0) |  |
| AFP |  |  | 0.002 |  |  | 0.078 |
| Negative | 523(31.4) | 191(25.5) |  | 229(30.7) | 190(25.5) |  |
| Positive | 751(45.1) | 394(52.7) |  | 362(48.6) | 393(52.8) |  |
| Borderline/Unknown | 392(23.5) | 163(21.8) |  | 154(20.7) | 162(21.7) |  |
| First Malignant |  |  |  |  |  | 0.677 |
| Yes | 1581(94.9) | 695(92.9) |  | 698(93.7) | 693(93.0) |  |
| No | 85(5.1) | 53(7.1) |  | 47(6.3) | 52(7.0) |  |
| Neoadjuvant Therapy |  |  | <0.001 |  |  | 0.916 |
| Yes | 482(28.9) | 308(41.2) |  | 302(40.5) | 305(40.9) |  |
| No | 1184(71.1) | 440(58.8) |  | 443(59.5) | 440(59.1) |  |
| Tumor Number |  |  | 0.010 |  |  | 0.835 |
| Single | 1038(62.3) | 424(56.7) |  | 417(56.0) | 422(56.6) |  |
| Multiple | 628(37.7) | 324(43.3) |  | 328(44.0) | 323(43.4) |  |
| Tumor Size |  |  | 0.300 |  |  | 0.748 |
| ≤28mm | 1032(61.9) | 447(59.8) |  | 456(61.2) | 445(59.7) |  |
| 28-95mm | 624(37.5) | 299(40.0) |  | 286(38.4) | 298(40.0) |  |
| >95mm | 10(0.6) | 2(0.3) |  | 3(0.4) | 2(0.3) |  |
| T Stage |  |  | 0.064 |  |  | 0.975 |
| T1a | 409(24.5) | 152(20.3) |  | 158(21.2) | 152(20.4) |  |
| T1b | 524(31.5) | 222(29.7) |  | 214(28.7) | 221(29.7) |  |
| T2 | 663(39.8) | 333(44.5) |  | 337(45.2) | 332(44.6) |  |
| T3 | 39(2.3) | 24(3.2) |  | 22(3.0) | 24(3.2) |  |
| T4 | 29(1.7) | 17(2.3) |  | 14(1.9) | 16(2.1) |  |
| TX | 2(0.1) | 0(0) |  | 0(0) | 0(0) |  |
| M Stage |  |  | 0.625 |  |  | 1.000 |
| M0 | 1659(99.6) | 743(99.3) |  | 740(99.3) | 741(99.5) |  |
| M1 | 7(0.4) | 5(0.7) |  | 5(0.7) | 4(0.5) |  |
| Grade^‡^ |  |  | <0.001 |  |  | 0.692 |
| G1-G2 | 1031(61.9) | 503(67.2) |  | 516(69.3) | 502(67.4) |  |
| G3-G4 | 135(8.1) | 80(10.7) |  | 70(9.4) | 78(10.5) |  |
| Unknown | 500(30.0) | 165(22.1) |  | 159(21.3) | 165(22.1) |  |
| Liver Cirrhosis |  |  | <0.001 |  |  | 0.424 |
| No | 73(4.4) | 54(7.2) |  | 42(5.6) | 54(7.2) |  |
| Yes | 625(37.5) | 326(43.6) |  | 336(45.1) | 324(43.5) |  |
| Unknown | 968(58.1) | 368(49.2) |  | 367(49.3) | 367(49.3) |  |
| 5yr-CSD | 0.127 | 0.138 | 0.957 | 0.125 | 0.137 | 0.828 |
| 5yr-OCSD | 0.117 | 0.100 | 0.624 | 0.107 | 0.100 | 0.463 |

LND=Lymph node dissection; PSM=Propensity score matching; HCC=Hepatocellular carcinoma; LT=Liver Transplantation; AFP=Alpha fetoprotein; CSD=Cancer-specific death; OCSD=Other cause-specific death.

^†^U.S. Census Bureau, Real Median Household Income in the United States [MEHOINUSA672N], retrieved from FRED, Federal Reserve Bank of St. Louis; https://fred.stlouisfed.org/series/MEHOINUSA672N, June 26, 2021

^‡^G1=Well differentiated; G2=Moderately differentiated; G3=Poorly differentiated; G4=Undifferentiated.

Table S6. Comparison between LND and Non-LND before and after PSM in pT1 HCC patients

| Factors | Before PSM | | | After PSM | | |
| --- | --- | --- | --- | --- | --- | --- |
|  | Non-LND  (n=4428) | LND  (n=643) | P | Non-LND  (n=640) | LND  (n=640) | P |
| Year of Diagnosis |  |  | 0.424 |  |  | 0.955 |
| 2004-2009 | 1871(42.3) | 283(44.0) |  | 280(43.8) | 282(44.1) |  |
| 2010-2015 | 2557(57.7) | 360(56.0) |  | 360(56.2) | 358(55.9) |  |
| Age |  |  | <0.001 |  |  | 0.919 |
| ≤72 | 3560(80.4) | 591(91.9) |  | 586(91.6) | 588(91.9) |  |
| >72 | 868(19.6) | 52(8.1) |  | 54(8.4) | 52(8.1) |  |
| Gender |  |  | 1.000 |  |  | 0.302 |
| Female | 1166(26.3) | 169(26.3) |  | 152(23.8) | 169(26.4) |  |
| Male | 3262(73.7) | 474(73.7) |  | 488(76.2) | 471(73.6) |  |
| Race |  |  | <0.001 |  |  | 0.965 |
| White | 2799(63.2) | 454(70.6) |  | 452(70.6) | 452(70.6) |  |
| Asia-Pacific | 996(22.5) | 94(14.6) |  | 89(13.9) | 94(14.7) |  |
| Black | 564(12.7) | 89(13.8) |  | 93(14.5) | 88(13.8) |  |
| Other | 69(1.6) | 6(0.9) |  | 6(0.9) | 6 (0.9) |  |
| Income^†^ |  |  | 0.370 |  |  | 0.648 |
| Below the median | 2530(57.1) | 380(59.1) |  | 389(60.8) | 380(59.4) |  |
| Above the median | 1898(42.9) | 263(40.9) |  | 251(39.2) | 260(40.6) |  |
| AFP |  |  | 0.759 |  |  | 0.477 |
| Negative | 1418(32.0) | 198(30.8) |  | 202(31.6) | 197(30.8) |  |
| Positive | 1953(44.1) | 293(45.6) |  | 271(42.3) | 291(45.5) |  |
| Borderline/Unknown | 1057(23.9) | 152(23.6) |  | 167(26.1) | 152(23.8) |  |
| First Malignant |  |  | 0.021 |  |  | 0.302 |
| Yes | 3800(85.8) | 574(89.3) |  | 583(91.1) | 571(89.2) |  |
| No | 628(14.2) | 69(10.7) |  | 57(8.9) | 69(10.8) |  |
| Neoadjuvant Therapy |  |  | <0.001 |  |  | 0.796 |
| Yes | 322(7.3) | 164(25.5) |  | 156(24.4) | 161(25.2) |  |
| No | 4106(92.7) | 479(74.5) |  | 484(75.6) | 479(74.8) |  |
| Tumor Number |  |  | - |  |  |  |
| Single | 4428(100.0) | 643(100.0) |  | 640(100.0) | 640(100.0) |  |
| Multiple | 0(0) | 0(0) |  | 0(0) | 0(0) |  |
| Tumor Size |  |  | 0.029 |  |  | 0.063 |
| ≤28mm | 1912(43.2) | 293(45.6) |  | 298(46.6) | 293(45.8) |  |
| 28-95mm | 2270(51.3) | 301(46.8) |  | 313(48.9) | 298(46.6) |  |
| >95mm | 246(5.6) | 49(7.6) |  | 29(4.5) | 49(7.7) |  |
| Surgery |  |  | <0.001 |  |  | 0.719 |
| LD | 1559(35.2) | 18(2.8) |  | 18(2.8) | 18(2.8) |  |
| LR | 1936(43.7) | 251(39.0) |  | 237(37.0) | 251(39.2) |  |
| LT | 933(21.1) | 374(58.2) |  | 385(60.2) | 371(58.0) |  |
| M Stage |  |  | 1.000 |  |  | 0.682 |
| M0 | 4391(99.2) | 638(99.2) |  | 638(99.7) | 636(99.4) |  |
| M1 | 37(0.8) | 5(0.8) |  | 2(0.3) | 4(0.6) |  |
| Grade^‡^ |  |  | 0.023 |  |  | 0.710 |
| G1-G2 | 2825(63.8) | 426(66.3) |  | 438(68.4) | 425(66.4) |  |
| G3-G4 | 512(11.6) | 88(13.7) |  | 78(12.2) | 86(13.4) |  |
| Unknown | 1091(24.6) | 129(20.1) |  | 124(19.4) | 129(20.2) |  |
| Liver Cirrhosis |  |  | <0.001 |  |  | 0.532 |
| No | 509(11.5) | 79(12.3) |  | 67(10.5) | 79(12.3) |  |
| Yes | 1026(23.2) | 204(31.7) |  | 199(31.1) | 201(31.4) |  |
| Unknown | 2893(65.3) | 360(56.0) |  | 374(58.4) | 360(56.2) |  |
| 5yr-CSD | 0.322 | 0.220 | <0.001 | 0.196 | 0.221 | 0.352 |
| 5yr-OCSD | 0.131 | 0.115 | 0.994 | 0.117 | 0.114 | 0.922 |

LND=Lymph node dissection; PSM=Propensity score matching; HCC=Hepatocellular carcinoma; AFP=Alpha fetoprotein; LD=Local destruction; LR=Liver resection; LT=Liver transplantation; CSD=Cancer-specific death; OCSD=Other cause-specific death.

^†^U.S. Census Bureau, Real Median Household Income in the United States [MEHOINUSA672N], retrieved from FRED, Federal Reserve Bank of St. Louis; https://fred.stlouisfed.org/series/MEHOINUSA672N, June 26, 2021

^‡^G1=Well differentiated; G2=Moderately differentiated; G3=Poorly differentiated; G4=Undifferentiated.

Table S7. Comparison between LND and Non-LND before and after PSM in pT2 HCC patients

| Factors | Before PSM | | | After PSM | | |
| --- | --- | --- | --- | --- | --- | --- |
|  | Non-LND  (n=2102) | LND  (n=481) | P | Non-LND  (n=640) | LND  (n=640) | P |
| Year of Diagnosis |  |  | 0.463 |  |  | 0.897 |
| 2004-2009 | 1038(49.4) | 228(47.4) |  | 231(48.0) | 228(47.4) |  |
| 2010-2015 | 1064(50.6) | 253(52.6) |  | 250(52.0) | 253(52.6) |  |
| Age |  |  | <0.001 |  |  | 0.774 |
| ≤72 | 1767(84.1) | 457(95.0) |  | 454(94.4) | 457(95.0) |  |
| >72 | 335(15.9) | 24(5.0) |  | 27(5.6) | 24(5.0) |  |
| Gender |  |  | 0.629 |  |  | 0.702 |
| Female | 474(22.5) | 114(23.7) |  | 108(22.5) | 114(23.7) |  |
| Male | 1628(77.5) | 367(76.3) |  | 373(77.5) | 367(76.3) |  |
| Race |  |  | <0.001 |  |  | 0.782 |
| White | 1416(67.4) | 359(74.6) |  | 367(76.3) | 359(74.6) |  |
| Asia-Pacific | 394(18.7) | 50(10.4) |  | 49(10.2) | 50(10.4) |  |
| Black | 260(12.4) | 67(13.9) |  | 58(12.1) | 67(13.9) |  |
| Other | 32(1.5) | 5(1.0) |  | 7(1.5) | 5(1.0) |  |
| Income^†^ |  |  | 0.173 |  |  | 1.000 |
| Below the median | 1224(58.2) | 297(61.7) |  | 298(62.0) | 297(61.7) |  |
| Above the median | 878(41.8) | 184(38.3) |  | 183(38.0) | 184(38.3) |  |
| AFP |  |  | 0.026 |  |  | 0.138 |
| Negative | 543(25.8) | 96(20.0) |  | 121(25.2) | 96(20.0) |  |
| Positive | 1135(54.0) | 278(57.8) |  | 254(52.8) | 278(57.8) |  |
| Borderline/Unknown | 424(20.2) | 107(22.2) |  | 106(22.0) | 107(22.2) |  |
| First Malignant |  |  | 0.035 |  |  | 0.913 |
| Yes | 283(13.5) | 47(9.8) |  | 45(9.4) | 47(9.8) |  |
| No | 1819(86.5) | 434(90.2) |  | 436(90.6) | 434(90.2) |  |
| Neoadjuvant Therapy |  |  | <0.001 |  |  | 0.491 |
| Yes | 262(12.5) | 150(31.2) |  | 161(33.5) | 150(31.2) |  |
| No | 1840(87.5) | 331(68.8) |  | 320(66.5) | 331(68.8) |  |
| Tumor Number |  |  | 0.152 |  |  | 0.830 |
| Single | 663(31.5) | 135(28.1) |  | 139(28.9) | 135(28.1) |  |
| Multiple | 1439(68.5) | 346(71.9) |  | 342(71.1) | 346(71.9) |  |
| Tumor Size |  |  | 0.002 |  |  | 0.323 |
| ≤28mm | 863(41.1) | 227(47.2) |  | 219(45.5) | 227(47.2) |  |
| 28-95mm | 1109(52.8) | 213(44.3) |  | 231(48.0) | 213(44.3) |  |
| >95mm | 130(6.2) | 41(8.5) |  | 31(6.4) | 41(8.5) |  |
| Surgery |  |  | <0.001 |  |  | 0.899 |
| LD | 641(30.5) | 11(2.3) |  | 10(2.1) | 11(2.3) |  |
| LR | 798(38.0) | 137(28.5) |  | 143(29.7) | 137(28.5) |  |
| LT | 663(31.5) | 333(69.2) |  | 328(68.2) | 333(69.2) |  |
| M Stage |  |  | 0.133 |  |  | 1.000 |
| M0 | 2067(98.3) | 478(99.4) |  | 479(99.6) | 478(99.4) |  |
| M1 | 35(1.7) | 3(0.6) |  | 2(0.4) | 3(0.6) |  |
| Grade^‡^ |  |  | <0.001 |  |  | 0.934 |
| G1-G2 | 1261(60.0) | 326(67.8) |  | 329(68.4) | 326(67.8) |  |
| G3-G4 | 297(14.1) | 85(17.7) |  | 86(17.9) | 85(17.7) |  |
| Unknown | 544(25.9) | 70(14.6) |  | 66(13.7) | 70(14.6) |  |
| Liver Cirrhosis |  |  | <0.001 |  |  | 0.611 |
| No | 199(9.5) | 50(10.4) |  | 41(8.5) | 50(10.4) |  |
| Yes | 564(26.8) | 177(36.8) |  | 180(37.4) | 177(36.8) |  |
| Unknown | 1339(63.7) | 254(52.8) |  | 260(54.1) | 254(52.8) |  |
| 5yr-CSD | 0.398 | 0.222 | <0.001 | 0.253 | 0.222 | 0.283 |
| 5yr-OCSD | 0.126 | 0.096 | 0.325 | 0.110 | 0.096 | 0.528 |

LND=Lymph node dissection; PSM=Propensity score matching; HCC=Hepatocellular carcinoma; AFP=Alpha fetoprotein; LD=Local destruction; LR=Liver resection; LT=Liver transplantation; CSD=Cancer-specific death; OCSD=Other cause-specific death.

^†^U.S. Census Bureau, Real Median Household Income in the United States [MEHOINUSA672N], retrieved from FRED, Federal Reserve Bank of St. Louis; https://fred.stlouisfed.org/series/MEHOINUSA672N, June 26, 2021

^‡^G1=Well differentiated; G2=Moderately differentiated; G3=Poorly differentiated; G4=Undifferentiated.

Table S8. Comparison between LND and Non-LND before and after PSM in pT3 HCC patients

| Factors | Before PSM | | | After PSM | | |
| --- | --- | --- | --- | --- | --- | --- |
|  | Non-LND  (n=536) | LND  (n=120) | P | Non-LND  (n=116) | LND  (n=116) | P |
| Year of Diagnosis |  |  | 0.588 |  |  | 0.793 |
| 2004-2009 | 272(50.7) | 57(47.5) |  | 59(50.9) | 56(48.3) |  |
| 2010-2015 | 264(49.3) | 63(52.5) |  | 57(49.1) | 60(51.7) |  |
| Age |  |  | 0.142 |  |  | 0.725 |
| ≤72 | 406(75.7) | 99(82.5) |  | 98(84.5) | 95(81.9) |  |
| >72 | 130(24.3) | 21(17.5) |  | 18(15.5) | 21(18.1) |  |
| Gender |  |  | 0.907 |  |  | 1.000 |
| Female | 133(24.8) | 31(25.8) |  | 30(25.9) | 31(26.7) |  |
| Male | 403(75.2) | 89(74.2) |  | 86(74.1) | 85(73.3) |  |
| Race |  |  | 0.351 |  |  | 0.144 |
| White | 345(64.4) | 70(58.3) |  | 81(69.8) | 68(58.6) |  |
| Asia-Pacific | 103(19.2) | 23(19.2) |  | 14(12.1) | 23(19.8) |  |
| Black | 77(14.4) | 25(20.8) |  | 21(18.1) | 23(19.8) |  |
| Other | 11(2.1) | 2(1.7) |  | 0(0) | 2(1.7) |  |
| Income^†^ |  |  | 0.458 |  |  | 0.168 |
| Below the median | 308(57.5) | 74(61.7) |  | 81(69.8) | 70(60.3) |  |
| Above the median | 228(42.5) | 46(38.3) |  | 35(30.2) | 46(39.7) |  |
| AFP |  |  | 0.535 |  |  | 0.988 |
| Negative | 115(21.5) | 30(25.0) |  | 31(26.7) | 30(25.9) |  |
| Positive | 297(55.4) | 67(55.8) |  | 62(53.4) | 63(54.3) |  |
| Borderline/Unknown | 124(23.1) | 23(19.2) |  | 23(19.8) | 23(19.8) |  |
| First Malignant |  |  | 0.133 |  |  | 0.815 |
| Yes | 456(85.1) | 109(90.8) |  | 107(92.2) | 105(90.5) |  |
| No | 80(14.9) | 11(9.2) |  | 9(7.8) | 11(9.5) |  |
| Neoadjuvant Therapy |  |  | 0.002 |  |  | 0.691 |
| Yes | 35(6.5) | 19(15.8) |  | 13(11.2) | 16(13.8) |  |
| No | 501(93.5) | 101(84.2) |  | 103(88.8) | 100(86.2) |  |
| Tumor Number |  |  | - |  |  | - |
| Single | 0(0) | 0(0) |  | 0(0) | 0(0) |  |
| Multiple | 536(100.0) | 120(100.0) |  | 116(100.0) | 116(100.0) |  |
| Tumor Size |  |  | 0.155 |  |  | 1.000 |
| ≤28mm | 0(0) | 0(0) |  | 0(0) | 0(0) |  |
| 28-95mm | 348(64.9) | 69(57.5) |  | 66(56.9) | 65(56.0) |  |
| >95mm | 188(35.1) | 51(42.5) |  | 50(43.1) | 51(44.0) |  |
| Surgery |  |  | <0.001 |  |  | 0.799 |
| LD | 152(28.4) | 1(0.8) |  | 1(0.9) | 1(0.9) |  |
| LR | 345(64.4) | 95(79.2) |  | 91(78.4) | 95(81.9) |  |
| LT | 39(7.3) | 24(20.0) |  | 24(20.7) | 20(17.2) |  |
| M Stage |  |  | 1.000 |  |  | 0.568 |
| M0 | 511(95.3) | 115(95.8) |  | 108(93.1) | 111(95.7) |  |
| M1 | 25(4.7) | 5(4.2) |  | 8(6.9) | 5(4.3) |  |
| Grade^‡^ |  |  | 0.202 |  |  | 0.702 |
| G1-G2 | 330(61.6) | 330(61.6) |  | 75(64.7) | 69(59.5) |  |
| G3-G4 | 111(20.7) | 32(26.7) |  | 27(23.3) | 32(27.6) |  |
| Unknown | 95(17.7) | 15(12.5) |  | 14(12.1) | 15(12.9) |  |
| Liver Cirrhosis |  |  | 0.299 |  |  | 0.687 |
| No | 75(14.0) | 23(19.2) |  | 21(18.1) | 23(19.8) |  |
| Yes | 92(17.2) | 22(18.3) |  | 17(14.7) | 21(18.1) |  |
| Unknown | 369(68.8) | 75(62.5) |  | 78(67.2) | 72(62.1) |  |
| 5yr-CSD | 0.677 | 0.588 | 0.138 | 0.619 | 0.606 | 0.780 |
| 5yr-OCSD | 0.089 | 0.094 | 0.838 | 0.062 | 0.097 | 0.590 |

LND=Lymph node dissection; PSM=Propensity score matching; HCC=Hepatocellular carcinoma; AFP=Alpha fetoprotein; LD=Local destruction; LR=Liver resection; LT=Liver transplantation; CSD=Cancer-specific death; OCSD=Other cause-specific death.

^†^U.S. Census Bureau, Real Median Household Income in the United States [MEHOINUSA672N], retrieved from FRED, Federal Reserve Bank of St. Louis; https://fred.stlouisfed.org/series/MEHOINUSA672N, June 26, 2021

^‡^G1=Well differentiated; G2=Moderately differentiated; G3=Poorly differentiated; G4=Undifferentiated.

Table S9. Comparison between LND and Non-LND before and after PSM in pT4 HCC patients

| Factors | Before PSM | | | After PSM | | |
| --- | --- | --- | --- | --- | --- | --- |
|  | Non-LND  (n=365) | LND  (n=102) | P | Non-LND  (n=97) | LND  (n=97) | P |
| Year of Diagnosis |  |  | 0.111 |  |  | 0.773 |
| 2004-2009 | 203(55.6) | 47(46.1) |  | 43(44.3) | 46(47.4) |  |
| 2010-2015 | 162(44.4) | 55(53.9) |  | 54(55.7) | 51(52.6) |  |
| Age |  |  | 0.311 |  |  | 0.687 |
| ≤72 | 289(79.2) | 86(84.3) |  | 84(86.6) | 81(83.5) |  |
| >72 | 76(20.8) | 16(15.7) |  | 13(13.4) | 16(16.5) |  |
| Gender |  |  | 0.181 |  |  | 0.627 |
| Female | 85(23.3) | 31(30.4) |  | 24(24.7) | 28(28.9) |  |
| Male | 280(76.7) | 71(69.6) |  | 73(75.3) | 69(71.1) |  |
| Race |  |  | 0.645 |  |  | 0.127 |
| White | 207(56.7) | 57(55.9) |  | 40(41.2) | 54(55.7) |  |
| Asia-Pacific | 97(26.6) | 30(29.4) |  | 40(41.2) | 29(29.9) |  |
| Black | 56(15.3) | 15(14.7) |  | 17(17.5) | 14(14.4) |  |
| Other | 5(1.4) | 0(0) |  | 0(0) | 0(0) |  |
| Income^†^ |  |  | 0.843 |  |  | 0.385 |
| Below the median | 212(58.1) | 61(59.8) |  | 51(52.6) | 58(59.8) |  |
| Above the median | 153(41.9) | 41(40.2) |  | 46(47.4) | 39(40.2) |  |
| AFP |  |  | 0.272 |  |  | 0.826 |
| Negative | 71(19.5) | 17(16.7) |  | 19(19.6) | 16(16.5) |  |
| Positive | 208(57.0) | 67(65.7) |  | 62(63.9) | 63(64.9) |  |
| Borderline/Unknown | 86(23.6) | 18(17.6) |  | 16(16.5) | 18(18.6) |  |
| First Malignant |  |  | 0.930 |  |  | 0.805 |
| Yes | 329(90.1) | 91(89.2) |  | 89(91.8) | 87(89.7) |  |
| No | 36(9.9) | 11(10.8) |  | 8(8.2) | 10(10.3) |  |
| Neoadjuvant Therapy |  |  | 0.005 |  |  | 0.833 |
| Yes | 23(6.3) | 16(15.7) |  | 85(87.6) | 83(85.6) |  |
| No | 342(93.7) | 86(84.3) |  | 12(12.4) | 14(14.4) |  |
| Tumor Number |  |  | 0.926 |  |  | 0.497 |
| Single | 322(88.2) | 89(87.3) |  | 88(90.7) | 84(86.6) |  |
| Multiple | 43(11.8) | 13(12.7) |  | 9(9.3) | 13(13.4) |  |
| Tumor Size |  |  | 0.381 |  |  | 0.945 |
| ≤28mm | 36(9.9) | 11(10.8) |  | 8(8.2) | 9(9.3) |  |
| 28-95mm | 182(49.9) | 43(42.2) |  | 41(42.3) | 42(43.3) |  |
| >95mm | 147(40.3) | 48(47.1) |  | 48(49.5) | 46(47.4) |  |
| Surgery |  |  | <0.001 |  |  | 0.920 |
| LD | 88(24.1) | 2(2.0) |  | 2(2.1) | 2(2.1) |  |
| LR | 248(67.9) | 83(81.4) |  | 82(84.5) | 80(82.5) |  |
| LT | 29(7.9) | 17(16.7) |  | 13(13.4) | 15(15.5) |  |
| M Stage |  |  | 0.541 |  |  | 0.805 |
| M0 | 335(91.8) | 91(89.2) |  | 87(89.7) | 89(91.8) |  |
| M1 | 30(8.2) | 11(10.8) |  | 10(10.3) | 8(8.2) |  |
| Grade^‡^ |  |  | 0.001 |  |  | 0.778 |
| G1-G2 | 163(44.7) | 50(49.0) |  | 51(52.6) | 48(49.5) |  |
| G3-G4 | 124(34.0) | 46(45.1) |  | 42(43.3) | 43(44.3) |  |
| Unknown | 78(21.4) | 6(5.9) |  | 4(4.1) | 6(6.2) |  |
| Liver Cirrhosis |  |  | 0.009 |  |  | 0.244 |
| No | 40(11.0) | 23(22.5) |  | 14(14.4) | 23(23.7) |  |
| Yes | 61(16.7) | 13(12.7) |  | 14(14.4) | 11(11.3) |  |
| Unknown | 264(72.3) | 66(64.7) |  | 69(71.1) | 63(64.9) |  |
| 5yr-CSD | 0.711 | 0.666 | 0.376 | 0.697 | 0.670 | 0.613 |
| 5yr-OCSD | 0.112 | 0.108 | 0.801 | 0.149 | 0.114 | 0.847 |

LND=Lymph node dissection; PSM=Propensity score matching; HCC=Hepatocellular carcinoma; AFP=Alpha fetoprotein; LD=Local destruction; LR=Liver resection; LT=Liver transplantation; CSD=Cancer-specific death; OCSD=Other cause-specific death.

^†^U.S. Census Bureau, Real Median Household Income in the United States [MEHOINUSA672N], retrieved from FRED, Federal Reserve Bank of St. Louis; https://fred.stlouisfed.org/series/MEHOINUSA672N, June 26, 2021

^‡^G1=Well differentiated; G2=Moderately differentiated; G3=Poorly differentiated; G4=Undifferentiated.

Table S10. Competing risk survival analyses of HCC patients with LND

| Factors | No. of Patients  (n=1287) | Univariable | | Multivariate | |
| --- | --- | --- | --- | --- | --- |
|  |  | P-CSD | P-OCSD | SHR (95%CI) | P |
| Year of Diagnosis |  | 0.237 | 0.128 |  |  |
| 2004-2009 | 576(44.8) |  |  | Reference |  |
| 2010-2015 | 711(55.2) |  |  | 0.924(0.751-1.136) | 0.450 |
| Age |  | <0.001 | 0.026 |  |  |
| ≤72 | 1182(91.8) |  |  | Reference |  |
| >72 | 105(8.2) |  |  | 1.075(0.775-1.491) | 0.670 |
| Gender |  | 0.212 | 0.178 |  |  |
| Female | 336(26.1) |  |  | Reference |  |
| Male | 951(73.9) |  |  | 1.164(0.916-1.479) | 0.210 |
| Race |  | 0.002 | 0.001 |  |  |
| White | 897(69.7) |  |  | Reference |  |
| Asia-Pacific | 189(14.7) |  |  | 1.070(0.807-1.418) | 0.640 |
| Black | 188(14.6) |  |  | 1.007(0.772-1.313) | 0.960 |
| Other | 13(1.0) |  |  | 1.398(0.470-4.159) | 0.550 |
| Income^†^ |  | 0.620 | 0.411 |  |  |
| Below the median | 780(60.6) |  |  | Reference |  |
| Above the median | 507(39.4) |  |  | 1.054(0.854-1.301) | 0.620 |
| AFP |  | 0.012 | 0.265 |  |  |
| Negative | 325(25.3) |  |  | Reference |  |
| Positive | 682(53.0) |  |  | 1.274(0.989-1.642) | 0.061 |
| Borderline/Unknown | 280(21.8) |  |  | 1.127(0.840-1.512) | 0.430 |
| First Malignant |  | 0.107 | 0.078 |  |  |
| Yes | 1161(90.2) |  |  | Reference |  |
| No | 126(9.8) |  |  | 1.061(0.753-1.494) | 0.740 |
| Neoadjuvant Therapy |  | <0.001 | 0.337 |  |  |
| Yes | 341(26.5) |  |  | Reference |  |
| No | 946(73.5) |  |  | 1.298(0.969-1.740) | 0.810 |
| Tumor Number |  | 0.548 | 0.204 |  |  |
| Single | 825(64.1) |  |  | Reference |  |
| Multiple | 462(35.9) |  |  | 0.895(0.617-1.299) | 0.560 |
| Tumor Size |  | <0.001 | 0.001 |  |  |
| ≤28mm | 515(40.0) |  |  | Reference |  |
| 28-95mm | 591(45.9) |  |  | 1.896(1.342-2.679) | <0.001 |
| >95mm | 181(14.1) |  |  | 2.691(1.764-4.105) | <0.001 |
| Surgery |  | <0.001 | 0.304 |  |  |
| LD | 23(1.8) |  |  | Reference |  |
| LR | 539(41.9) |  |  | 0.570(0.358-0.909) | 0.018 |
| LT | 725(56.3) |  |  | 0.238(0.149-0.982) | <0.001 |
| No. of Lymph Node Retrieved |  | 0.890 | 0.668 |  |  |
| 1 | 748(58.1) |  |  | Reference |  |
| 2 | 265(20.6) |  |  | 1.212(0.937-1.567) | 0.140 |
| 3 | 139(10.8) |  |  | 1.153(0.848-1.567) | 0.360 |
| ≥4 | 135(10.5) |  |  | 0.719(0.482-1.072) | 0.110 |
| T Stage |  | <0.001 | 0.031 |  |  |
| T1a | 175(13.6) |  |  | Reference |  |
| T1b | 437(34.0) |  |  | 0.998(0.581-1.713) | 0.990 |
| T2 | 460(35.7) |  |  | 1.207(0.678-2.150) | 0.520 |
| T3 | 116(9.0) |  |  | 2.188(0.990-4.836) | 0.053 |
| T4 | 99(7.7) |  |  | 2.218(1.184-4.155) | 0.130 |
| N Stage |  | <0.001 | 0.167 |  |  |
| N0 | 1234(95.9) |  |  | Reference |  |
| N1 | 53(4.1) |  |  | 1.854(1.220-2.818) | 0.004 |
| M Stage |  | <0.001 | 0.276 |  |  |
| M0 | 1265(98.3) |  |  | Reference |  |
| M1 | 22(1.7) |  |  | 1.593(0.893-2.841) | 0.110 |
| Grade^‡^ |  | <0.001 | 0.127 |  |  |
| G1-G2 | 838(65.1) |  |  | Reference |  |
| G3-G4 | 243(18.9) |  |  | 1.568(1.218-2.018) | <0.001 |
| Unknown | 206(16.0) |  |  | 1.356(0.996-1.845) | 0.053 |
| Liver Cirrhosis |  | <0.001 | 0.074 |  |  |
| No | 171(13.3) |  |  | Reference |  |
| Yes | 402(31.2) |  |  | 1.606(1.125-2.292) | 0.009 |
| Unknown | 714(55.5) |  |  | 1.599(1.199-2.132) | 0.001 |

HCC=Hepatocellular carcinoma; LND=Lymph node dissection; AFP=Alpha fetoprotein; LD=Local destruction; LR=Liver resection; LT=Liver transplantation; CSD=Cancer-specific death; OCSD=Other cause-specific death; SHR= Subdistribution hazard ratio; CI=Confidence interval.

^†^U.S. Census Bureau, Real Median Household Income in the United States [MEHOINUSA672N], retrieved from FRED, Federal Reserve Bank of St. Louis; https://fred.stlouisfed.org/series/MEHOINUSA672N, June 26, 2021

^‡^G1=Well differentiated; G2=Moderately differentiated; G3=Poorly differentiated; G4=Undifferentiated.

Table S11. Baseline characteristics of HCC patients with LND in the training and validation set

| Factors | Training Set  (n=673) | Validation Set  (n=673) | P |
| --- | --- | --- | --- |
| Year of Diagnosis |  |  | 0.325 |
| 2004-2009 | 298(44.3) | 317(47.1) |  |
| 2010-2015 | 375(55.7) | 356(52.9) |  |
| Age |  |  | 0.913 |
| ≤58 | 314(46.7) | 311(46.2) |  |
| >58 | 359(53.3) | 362(53.8) |  |
| Gender |  |  | 0.318 |
| Female | 181(26.9) | 164(24.4) |  |
| Male | 492(73.1) | 509(75.6) |  |
| Race |  |  | 0.859 |
| White | 468(69.5) | 472(70.1) |  |
| Other | 205(30.5) | 201(29.9) |  |
| Income^†^ |  |  | 0.344 |
| Below the median | 397(59.0) | 415(61.7) |  |
| Above the median | 276(41.0) | 258(38.3) |  |
| AFP |  |  | 0.148 |
| Negative | 155(23.0) | 186(27.6) |  |
| Positive | 365(54.2) | 340(50.5) |  |
| Borderline/Unknown | 153(22.7) | 147(21.8) |  |
| First Malignant |  |  | 0.787 |
| Yes | 606(90.0) | 602(89.5) |  |
| No | 67(10.0) | 71(10.5) |  |
| Neoadjuvant Therapy |  |  | 0.619 |
| Yes | 179(26.6) | 170(25.3) |  |
| No | 494(73.4) | 503(74.7) |  |
| Tumor Number |  |  | 0.569 |
| Single | 428(63.6) | 439(65.2) |  |
| Multiple | 245(36.4) | 234(34.8) |  |
| Tumor Size |  |  | 0.278 |
| ≤64mm | 520(77.3) | 502(74.6) |  |
| >64mm | 153(22.7) | 171(25.4) |  |
| T Stage |  |  | 1.000 |
| T1-2 | 562(83.5) | 562(83.5) |  |
| T3-4 | 111(16.5) | 111(16.5) |  |
| Intrahepatic Vascular Invasion |  |  | 0.051 |
| No/Unknown | 581(86.3) | 554(82.3) |  |
| Yes | 92(13.7) | 119(17.7) |  |
| Major Vascular Invasion |  |  | 0.302 |
| No/Unknown | 637(94.7) | 646(96.0) |  |
| Yes | 36(5.3) | 27(4.0) |  |
| Gallbladder Invasion Only |  |  | 1.000 |
| No/Unknown | 663(98.5) | 663(98.5) |  |
| Yes | 10(1.5) | 10(1.5) |  |
| Extrahepatic Bile Duct Invasion^‡^ |  |  | 0.552 |
| No/Unknown | 662(98.4) | 658(97.8) |  |
| Yes | 11(1.6) | 15(2.2) |  |
| Visceral Peritoneum Invasion |  |  | 1.000 |
| No/Unknown | 656(97.5) | 655(97.3) |  |
| Yes | 17(2.5) | 18(2.7) |  |
| Peripheral Organ Invasion |  |  | 1.000 |
| No/Unknown | 661(98.2) | 662(98.3) |  |
| Yes | 12(1.8) | 11(1.6) |  |
| Grade^§^ |  |  | 0.759 |
| G1-G2 | 431(64.0) | 444(66.0) |  |
| G3-G4 | 129(19.2) | 122(18.1) |  |
| Unknown | 113(16.8) | 107(15.9) |  |
| Liver Cirrhosis |  |  | 0.387 |
| No | 96(14.3) | 79(11.7) |  |
| Yes | 205(30.5) | 211(31.4) |  |
| Unknown | 372(55.3) | 383(56.9) |  |
| RLNM |  |  | 0.339 |
| No | 649(96.4) | 641(95.2) |  |
| Yes | 24(3.6) | 32(4.8) |  |

HCC=Hepatocellular carcinoma; LND=Lymph node dissection; AFP=Alpha fetoprotein; RLNM=Regional Lymph Node Metastasis.

^†^U.S. Census Bureau, Real Median Household Income in the United States [MEHOINUSA672N], retrieved from FRED, Federal Reserve Bank of St. Louis; https://fred.stlouisfed.org/series/MEHOINUSA672N, June 26, 2021

^‡^Including gallbladder invasion

^§^G1=Well differentiated; G2=Moderately differentiated; G3=Poorly differentiated; G4=Undifferentiated

**Table S12**. Coefficient for each factor in the LASSO regression models

| Factors | Coefficient |
| --- | --- |
| Year of Diagnosis | - |
| Age | - |
| Gender | -0.12919967 |
| Race | 0.18959773 |
| Income | - |
| AFP | - |
| First Malignant | - |
| Neoadjuvant Therapy | - |
| Tumor Number | - |
| Tumor Size | 1.05256304 |
| cT Stage | 0.79920286 |
| Intrahepatic Vascular Invasion | - |
| Major Vascular Invasion | 0.06175452 |
| Gallbladder Invasion Only | - |
| Extrahepatic Bile Duct Invasion | 1.31712011 |
| Visceral Peritoneum Invasion | - |
| Peripheral Organ Invasion | - |
| Grade | 0.02345511 |
| Liver Cirrhosis | - |

LASSO=Least absolute shrinkage and selection operator; AFP=Alpha fetoprotein.
